# Targeted delivery of miR-99b reprograms tumor-associated macrophage phenotype leading to tumor regression

**DOI:** 10.1136/jitc-2019-000517

**Published:** 2020-09-18

**Authors:** Liang Wang, Yi-Yang Hu, Jun-Long Zhao, Fei Huang, Shi-Qian Liang, Lei Dong, Yan Chen, Heng-Chao Yu, Jian Bai, Jia-Meng Yang, Jie-Yi Fan, Lei Feng, San-Zhong Li, Hua Han, Hong-Yan Qin

**Affiliations:** 1 State Key Laboratory of Cancer Biology, Department of Medical Genetics and Developmental Biology, Fourth Military Medical University, Xi'an, Shaanxi, China; 2 State Key Laboratory of Pharmaceutical Biotechnology, School of Life Sciences, Nanjing University, Nanjing, Jiangsu, China; 3 Department of Clinical Oncology, Xijing Hospital, Fourth Military Medical University, Xi'an, Shaanxi, China; 4 Department of Hepatobiliary Surgery, Xijing Hospital, Fourth Military Medical University, Xi'an, Shaanxi, China; 5 State Key Laboratory of Cancer Biology, Department of Biochemistry and Molecular Biology, Fourth Military Medical University, Xi'an, Shaanxi, China

**Keywords:** immunotherapy, antigen presentation, phagocytosis, liver neoplasms, macrophages

## Abstract

**Background:**

Accumulating evidence has shown that tumor-associated macrophages (TAMs) play a critical role in tumor progression. Targeting TAMs is a potential strategy for tumor immunotherapy. However, the mechanism underlying the TAM phenotype and function needs to be resolved. Our previous studies have demonstrated that miR-125a can reverse the TAM phenotype toward antitumor. Meanwhile, we have found that miR-125a and miR-99b cluster in the first intron of the same host gene, and are transcribed simultaneously in bone marrow-derived macrophages (BMDMs) following LPS+IFNγ stimulation. However, it remains unclear whether miR-99b by itself can exert an antitumor effect by regulating macrophage phenotype.

**Methods:**

miR-99b and/or miR-125a were delivered into TAMs of orthotopic hepatocellular carcinoma (HCC) or subcutaneous Lewis lung cancer (LLC) mice. The effect of treatment was evaluated by live imaging, TUNEL staining and survival tests. The phenotype of the immune cells was determined by qRT-PCR, ELISA, western blot and FACS. The capability of miR-99b-mediated macrophage phagocytosis and antigen presentation was detected by FACS and immunofluorescence staining. The underlying molecular mechanism was examined by qRT-PCR, reporter assay and western blot, and further verified in the tumor model. The expression of miR-99b and its target genes was determined in TAMs sorted from tumor and adjacent tissues in patients with liver cancer.

**Results:**

Targeted delivery of miR-99b and/or miR-125a into TAMs significantly impeded the growth of HCC and LLC, especially after miR-99b delivery. More importantly, the delivery of miR-99b re-educated TAM toward antitumor phenotype with enhanced immune surveillance. Further investigation of mechanisms showed that macrophage-specific overexpression of miR-99b promoted M1 while suppressing M2 macrophage polarization by targeting κB-Ras2 and/or mTOR, respectively. miR-99b-overexpressed M1 macrophage was characterized by stronger capability of phagocytosis and antigen presentation. Additionally, delivery of simTOR or siκB-Ras2 into TAMs inhibited miR-99b antagomir-triggered tumor growth. Finally, miR-99b expression was lower in TAMs of patients with liver cancer than that in adjacent tissues, while the expression of κB-Ras2 and mTOR was reversed.

**Conclusions:**

Our results reveal the mechanism of miR-99b-mediated TAM phenotype, indicating that TAM-targeted delivery of miR-99b is a potential strategy for cancer immunotherapy.

## Background

Tumor-associated macrophages (TAMs) play a vital role in tumor microenvironment (TME) by mediating immunosuppression, promoting angiogenesis and tumor metastasis and even exerting drug resistance.^1–3^ Based on the environmental cues, TAMs can exhibit tumoricidal M1-tumor or pro-tumor M2-like macrophage phenotype. On IL-4 or IL-13 stimulation, TAMs differentiate into M2-like TAM phenotype that can suppress T-cell activation and proliferation and promote angiogenesis by secreting IL-10, TGF-β and a large amount of proangiogenic factors.^2 4^ Clinical studies show that poor prognosis of cancer patients is highly correlated with the number of M2-like TAMs.^5^ In contrast, LPS and/or IFNγ induction results in M1-like TAMs that exhibit antitumor capacity by producing IL-12, TNFα and iNOS as well as initiating Th1 response, which is associated with good prognosis of cancer patients.^4 5^ Owing to the divergent roles of TAMs in TME, instead of directly targeting tumor cells, modulating TAMs is an emerging strategy of great potential and value for the treatment of solid tumors. Currently, the immunotherapy strategies target TAMs primarily by interfering with M2-like TAM survival, blocking macrophage recruitment and promoting M2-like to M1-like TAM switch.^6^ However, the mechanism underlying TAM polarization and activation needs to be further investigated.

Accumulating evidence including our previous studies has demonstrated that microRNAs (miRNAs) participate in myeloid differentiation, macrophage activation and tumor immunity by suppressing the expression of target genes.^7–10^ Several studies reported that miR-99b can modulate the immune response during pathogen infection and chronic inflammation.^11 12^ Recently, it was shown that miR-99b/let-7e/125a and miR-212/132, two miRNA clusters, can promote monocyte-to-osteoclast differentiation directed by NF-κB signaling.^13^ The set of miRNAs, including miR-99b, is responsible for the conversion of monocytes into myeloid-derived suppressor cells (MDSCs) and resistance to immunotherapy in melanoma patients.^14^ In line with these findings, our group also identified a series of miRNA including miR-125a, miR-148a and miR-99b as downstream genes of Notch signaling in LPS-stimulated macrophages. miR-125a could promote M1 while repressing M2 macrophage polarization, leading to tumor regression by reprogramming the antitumor immune microenvironment.^7 8^ Furthermore, miR-125a and miR-99b were found to cluster in the first intron of the same host gene *Spaca6A*, and were transcribed simultaneously in bone marrow-derived macropahges (BMDMs) following LPS+IFNγ stimulation (online supplementary figure S1A and B).^7^ However, it remains to be determined whether miR-99b can exert similar to miR-125a antitumor effect by regulating macrophage polarization.

10.1136/jitc-2019-000517.supp1 Supplementary data

Re-educating M2-like TAMs toward an antitumor M1-like phenotype has been regarded as a promising strategy for cancer treatment.^2 6 15 16^ Using genetically engineered mice bearing pancreatic cancer, Beatty *et al* unexpectedly found that administration of an agonistic anti-CD40 antibody could re-educate M2-like toward M1-like TAMs with enhanced antigen-presenting capabilities, leading to tumor immune surveillance recovery and tumor regression.^17^ In a preclinical model of pancreatic cancer, administration of PI3Kγ inhibitor or Bruton’s tyrosine kinase inhibitor resets TAMs toward antitumor M1 phenotype following by the reduction in tumor volume.^18^ In addition, repolarization of M2-like toward M1-like macrophages has been achieved in mice by inhibiting class IIa histone deacetylases, overexpressing histone-rich glycoprotein or enhancing TLR7/TLR8 signaling.^19–21^To ensure specific TAM targeting, a TAM-targeted delivery system has recently been developed. Tesz *et al* successfully engineered glucan particles for selective delivery of siRNA to phagocytic macrophages in cultured cells or mice.^22^ Subsequently, Huang *et al* established a nucleic acid delivery system to target TAMs or tumor-infiltrating dendritic cells (TIDCs) to treat liver and breast cancer. These studies show that targeted delivery of polymeric nanoparticles loaded with certain siRNA or miRNA can reprogram the TAM phenotype and abrogate tumor growth.^23 24^ Therefore, targeted delivery of miRNAs to TAMs and re-educating TAMs represent a promising immunotherapy for cancer. However, effective endogenous miRNAs that could regulate macrophage repolarisation remain to be explored.

In the present study, we demonstrated that TAM-targeted delivery of miR-99b and/or miR-125a using Huang’s drug delivery system could significantly impede the growth of murine hepatocellular carcinoma (HCC) or Lewis lung cancer (LLC). Further studies of cellular mechanisms showed that delivery of miR-99b and/or miR-125a into TAMs repolarized M2-like TAMs toward the M1 phenotype and reprogrammed the antitumor immune microenvironment. Moreover, the molecular mechanism revealed that miR-99b might promote M1 while inhibiting M2 polarization by downregulating κB-Ras2 and/or mTOR, respectively. Interestingly, miR-99b might amplify M1 macrophage function through NF-κB by a positive feedback regulation loop, resulting in increased phagocytosis and antigen presentation. Moreover, delivery of miR-99b antagomir and siRNA against mTOR or κB-Ras2 into TAMs reduced tumor growth compared with miR-99b antagomir delivery. Finally, in clinical samples, we found that the expression of miR-99b and miR-125a was lower in human TAMs of liver cancer than that in adjacent tissues, while the expression of κB-Ras2 and mTOR was reversed, indicating that the axis of miR-99b/mTOR and/or κB-ras2 also participated in regulation of the TAM phenotype in patients with liver cancer.

In summary, our current study revealed that TAM-targeted delivery of miR-99b inhibited tumor growth by re-educating the TAM phenotype from protumor to antitumor and reprogramming the antitumor immune microenvironment. Thus, it has put forward a new candidate approach for cancer immunotherapy.

## Methods

### Patients and biopsies

Human HCC was obtained from hospitalized patients in the Department of Hepatobiliary Surgery, Xijing Hospital of Fourth Military Medical University, and staged according to the AJCC Cancer Staging Manual (eighth ed.) (online supplementary table S1).

10.1136/jitc-2019-000517.supp2 Supplementary data

### Mice and tumor models

Mice with C57BL/6 background were maintained in a specific-pathogen-free facility.

HCC cells (Hepa1-6) and LLC cells were purchased from the authenticated ATCC repository in 2016 and 2014, respectively. C57BL derivation and absence of mycoplasma contamination were confirmed. For the orthotopic tumor model, Hepa1-6 cells (2×10^6^ cells in 20 µL Matrigel, Sigma, St. Louis, MO) were injected into the left lobes of the livers of 6-week to 8-week-old C57BL/6 mice. For the subcutaneous tumor model, 2×10^6^ LLC cells were injected subcutaneously on the rear back of C57BL/6 mice. Tumor growth was monitored using an IVIS imaging system (Xenogen, Perkin-Elmer).

### Immunofluorescence and flow cytometry

Tumor sections or cells were stained with antibodies listed in online supplementary table S2, and observed under a laser scanning confocal microscope (FV-1000, Olympus, Tokyo). Apoptotic cells were detected using TUNEL assay kit (Promega, Madison, WI) according to the supplier’s instructions. Cell suspensions were incubated with primary antibodies and secondary antibodies (online supplementary table S2), and then analyzed using FACSCantoII (BD Biosciences, San Jose, CA) and sorted using SONY SH800 Automated Cell Sorter (SONY, Tokyo), respectively. Dead cells were excluded by seven-AAD staining. Intracellular staining was performed as described previously.^25^ Data were analyzed using Flowjo V.10 software (TreeStar, Ashland, OR).

Murine bone marrow (BM)-derived monocytes were isolated by magnetic-activated cell sorting (MACS) as previously described.^7^ For human TAM isolation, the surgical resection tumor and adjacent tissues in patients with HCC were digested with human tumor dissociation kit (Miltenyi Biotec, Bergisch Gladbach, Germany). Non-parenchymal cells were obtained by Percol (Solarbio, Beijing, China) density centrifugation as described previously.^26^ Subsequently, CD163^+^TAMs were sorted by MACS. The purity of murine CD11b^+^monocytes and human CD14^+^CD163^+^TAMs was further confirmed by FACS.

### TAM-targeted nucleic acid drug delivery system

Cationic konjac polysaccharide (cKGM) and PEG-His-modified alginate (PHA) were prepared as saline solutions as previously reported.^23 24^ The agomir of miR-99b, miR-125a, simTOR, siκB-Ras2 and their control, as well as the antagomir of miR-99b and control were purchased from RiboBio Biotech (Guangzhou, China). The cKGM nucleic acid complex was formed by mixing 5 mg/mL of agomir or antagomir with 5 mg/mL of cKGM (1:3). The formed complex was mixed with 5 mg/mL PHA (1:1) in order to form the triple complex (vector and miRNA). Endocytosis of cKGM and nucleic acid complex were determined by immunofluorescence, and the biodistribution of the nucleic acid drug was also detected as previously reported.^24^

### Antitumor assay of TAMs-targeted miRNA delivery system in tumor models

Tumor-bearing mice were intravenously injected with vector and miRNA with 2 µg miRNA/g body weight every 3 days from day 14 (HCC) or day 7 (LLC) after the injection of tumor cells. The tumor tissues were harvested at day 29 (HCC) or day 22 (LLC) for further analysis. For survival analysis, tumor-bearing mice were treated with nucleic acid drugs as mentioned above and then observed until day 120.

### Cell culture and transfection

BMDMs were obtained and cultured as previously described.^25^ Raw 264.7 cells were cultured in Dulbecco’s modified Eagle’s medium (DMEM) with 10% fetal bovine serum (FBS) and 2 mmol/L L-glutamine. BMDMs or Raw 264.7 cells were stimulated with LPS (50 ng/mL, Sigma, St. Louis, MO, USA) and IFNγ (20 ng/mL, PeproTech, Rocky Hill, USA) or IL-4 (20 ng/mL, PeproTech) for 24 hours to induce M1 or M2 polarized macrophages. γ-secretase inhibitor IX (GSI, 30 µM, Sigma), BAY11-7082 (5 µM, Selleck), IKK-16 (0.5 µM, MedChem Express) or Torin 1 (100 nM, MedChem Express) was added to the medium, with DMSO as a control. The THP1 cells (ATCC) were cultured at 2×10^5^ cells/mL in RPMI 1640 medium supplemented with 10% FBS and 2 mmol/L L-glutamine, differentiating into macrophages with 200 nM phorbol 12-myristate 13-acetate (PMA, Sigma) treatment for 2 days. The cells were transfected with the respective oligos using Lipofectamine 2000 reagent (Invitrogen, Waltham, MA, USA), according to the manufacture’s protocol. The sequences of siRNAs against mTOR, NF-κB inhibitor κB-Ras2 (also known as Nkiras2) and IRF4 were shown in online supplementary table S3.

### Phagocytosis assay

For flow cytometry, tumor cells were labeled with carboxyfluorescein diacetate succinimidyl ester (CFSE, Sigma, St. Louis, MO, USA), and cocultured with BMDMs transfected with miR-99b, antisense oligonucleotides (ASO) or control oligo, at a ratio of 400 000 tumor cells to 200 000 macrophages in ultra-low-attachment 24-well plates (Corning, Corning, NY) in serum-free DMEM. After 2 hours, the cocultured cells were harvested and stained with APC-labeled anti-F4/80 antibodies. The percentage of F4/80^+^CFSE^+^ cells in total F4/80^+^ macrophages was measured for phagocytosis analysis using FACScalibur (BD).

For the confocal microscope, BMDMs were added to the coverslips and allowed to adhere for 1 hour at 37°C. Tumor cells were labeled with the membrane dye Dil (Beyotime, Shanghai, China), and cocultured with adherent BMDMs for 6 hours. Following vigorous washing with serum-free DMEM, cells were stained with anti-F4/80 antibodies and Hoechst, and then observed under a confocal microscope (FV1000, Olympus).

### Mixed lymphocyte reaction assay

Mixed lymphocyte reaction assay was performed as previously described.^25^

### Bioinformatics analysis

The target genes of miR-99b were predicted using miRNA target gene prediction databases including DIANA, MIRDB, Pictar, RNA22 and Targetscan. In silico functional analysis of miR-99b in natural killer cells was performed using gene set enrichment analysis.^27^ Data from Gene Expression Omnibus were under the accession number GSE69555.^12^

### Reporter assay

The 3′-UTR of mTOR and κB-Ras2 was amplified using a mouse cDNA library as a template and mutated by PCR-based methods. The PCR primers were listed in online supplementary table S3. Wild type or mutant 3′-UTR fragments were inserted into the pGL3-promoter (Promega, Madison, WI, USA) to generate reporter plasmids. HEK293 cells were seeded in 48-well plates and transfected with different combinations of reporters and miRNA using Lipofectamine 2000 reagent, with a Renilla luciferase vector as an internal control. The cells were harvested 24 hours after transfection, and the relative luciferase activity was measured using the Dual Luciferase Reporter Assay System (Promega).

### Enzyme-linked immunosorbent assay

The amount of IL-12, TNFα, IL-6, IL-10 and IFNγ in the serum or the supernatant of cultured BMDMs with different stimuli was determined using an ELISA Ready-SET-Go kit (eBioscience) according to the supplier’s protocol.

### qRT-PCR

Total RNA extraction, reverse transcription and qRT-PCR were performed as described previously,^7^ with U6 RNA (for miRNAs) or β-actin as internal controls. The primers were shown in online supplementary table S3.

### Western blot

Cells were lysed in the RIPA buffer supplemented with protease inhibitors (Beyotime, Shanghai, China), and then nucleic and cytoplasmic proteins were extracted using the Extraction Kit (Beyotime, Shanghai, China) according to the manufacturer’s instructions. Protein concentrations were determined with BCA Protein Assay kit (Pierce, Waltham, MA, USA). Samples were separated by SDS-PAGE and blotted on polyvinylidenefluoride membranes. Membranes were blocked with 5% skim milk solution for 1 hour and then probed with primary antibodies and secondary antibodies, as listed in online supplementary table S2. Protein blots were developed using an ECL detection system (Pierce).

### Statistics

Images were quantitatively analyzed using Image Pro Plus V.6.0 software (MediaCybernetics Inc., Bethesda, MD, USA). Data were analyzed using Graph Pad Prism software (V. 5.0). Statistical significance was assessed with unpaired student’s t-test, paired t-test or one-way analysis of variance with Turkey’s multiple comparison tests. Survival curves were tested by the Kaplan-Meier method and statistical significance was determined by the log-rank (Mantel-Cox) test. P<0.05 was considered statistically significant.

## Results

### Validation of the TAM-targeted delivery of miR-99b or miR-125a system

miR-125a and miR-99b clustering in *Spaca6A* and simultaneous transcription in BMDMs under LPS+IFNγ stimulation were shown in online supplementary figure S1A and B. The expression of miR-99b was significantly increased following with murine or human monocyte differentiation (online supplementary figure S2A‒C). Overexpressed miR-99b in BM cells or monocytes promoted macrophage differentiation and maturation along with high expression of F4/80, MHCII and Vcam1 and low expression of Ly6C (online supplementary figure S2D, E), resembling the phenotype of miR-125a during myeloid development.^7^ Interestingly, we found that the expression of miR-99b and miR-125a was lower in sorted CD14^+^CD163^+^ TAMs of patients with liver cancer than in adjacent tissues (online supplementary figure S3). Therefore, we wondered whether transfection of miR-99b into TAMs could have the same effect on tumor growth as miR-125a.

Recently, Huang *et al* successfully constructed a nucleic acid drug delivery system that could target both TAMs and TIDCs.^24^ Taking advantage of this system, we conjugated miR-99b and/or miR-125a to this delivery system and observed their impact on tumorigenesis (online supplementary figure S4A). First, using immunofluorescence staining, we verified that miR-99b could be specifically delivered into BMDMs but not Hepa1-6 cells (online supplementary figure S4B). Second, administration with vector-loaded Cy5-labeled miR-99b (V&Cy5-miR-99b) by tail vein, in vivo live imaging showed that V&Cy5-miR-99b was enriched mostly in HCC rather than in other organs (online supplementary figure S4c). Finally, V&Cy5-miR-99b delivery could target F4/80^+^ TAMs as shown by immunofluorescence staining and FACS assay, respectively (online supplementary figure S4D and E). Collectively, we verified that this drug system could deliver miRNAs specifically into TAMs of HCC.

### TAM-targeted delivery of miR-99b and/or miR-125a inhibited tumor growth

**Figure 1:**
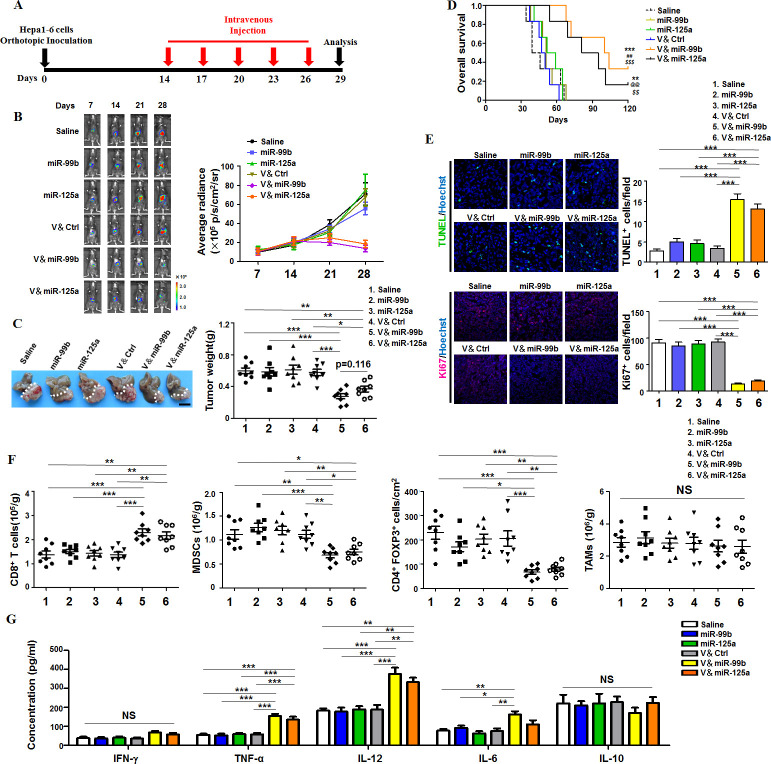
TAM-targeted delivery of miR-99b or miR-125a inhibited orthotopic HCC growth. (A) Schedule of HCC therapy by TAM-targeted delivery of miR-99b or miR-125a or control (Ctrl) agomir via mouse tail vein. HCC model was established by orthotopic hepatic inoculating Hepa1-6 cells that carry luciferase expression. (B) HCC-bearing mice were treated with different drugs according to the schedule as shown in (A). The HCC growth was monitored at different time points under a Xenogen IVIS system after intraperitoneal injection with luciferin. Average radiance was compared among each group (n=8). (C) Tumors were dissected and photographed at day 29 after treatment with different drugs as shown in (B). Tumor weight were measured and compared (n=8). (D) The survival curves of tumour-bearing mice were observed after treatment with different drugs as shown in (B). **p<0.01 and ***p<0.001 (vs saline); ^##^p<0.01 (vs miR-99b); ^$$^p<0.01 and ^$$$^p<0.001 (vs V＆Ctrl); ^@@^p<0.01 (vs miR-125a) using log-rank (Mantel-Cox) test. (E) HCC apoptosis and proliferation were detected using TUNEL (upper panel) and Ki67 staining (lower panel) after treatment with different drugs as shown in (B) (n=8). (F) The absolute immune cell numbers of HCC was calculated after FACS assay or histology immunofluorescence staining, including CD8^+^T cells, MDSCs, Treg cells and TAMs (n=8). (G) Serum from tumour-bearing mice with different drug treatment was collected and the concentration of the indicated cytokines was determined by ELISA (n=4). Data are shown as mean±SEM. * p<0.05; **p<0.01; ***p<0.001 using one-way ANOVA with Tukey’s multiple comparison test (C, E–G). ANOVA, analysis of variance; HCC, hepatocellular carcinoma; MDSCs, myeloid-derived suppressor cells; TAM, tumor-associated macrophage.

Next, we established the orthotopic HCC tumors and observed tumor growth using live imaging system (figure 1A, B). The result showed that the luciferase intensity of HCC was significantly weaker in both vector & miR-99b (V&miR-99b)-treated and vector & miR-125a (V&miR-125a)-treated mice than in other drugs-treated mice since day 21 (figure 1B). Consistently, tumor weight in both V&miR-99b-treated and V&miR-125a-treated mice were lighter than those in the other drug-treated mice (figure 1C). The survival curve indicated that 16.67%–33.33% of tumor-bearing mice treated with V&miR-99b or V&miR-125a remained alive until day 120, while the mice with naked miRNAs or saline administration died at earlier time (figure 1D). Immunofluorescence staining with TUNEL showed that the number of apoptotic HCC cells in both V&miR-99b-treated and V&miR-125a-treated mice were significantly more than those in other drug-treated mice. Correspondingly, the proliferation of HCC cells was remarkably reduced after V&miR-99b and V&miR-125a delivery (figure 1E). Furthermore, FACS assay showed higher number of CD8^+^T cells rather than CD4^+^T cells and decreased number of immunosuppressive cells including MDSCs and Treg cells (figure 1F and online supplementary figure S5A‒D). However, the total TAM number in HCC remained the same among different drug-treated tumor-bearing mice (figure 1F and online supplementary figure S5E), suggesting that the function of TAMs might change after delivery of miR-99b or miR-125a. Indeed, ELISA results indicated that the expression of serum TNFα, IL-12 and IL-6, which are functional markers of M1 macrophages, significantly increased in V&miR-99b-treated or V&miR-125a-treated tumor-bearing mice (figure 1G). Unexpectedly, the antitumor ability of TAM-targeted codelivery of miR-125a and miR-99b was less efficient than only miR-99b delivery (online supplementary figure S6). Moreover, the antitumor ability of TAM-targeted miR-99b or miR-125a delivery was also recapitulated in subcutaneous transplanted LLC tumor (online supplementary figure S7). Taken together, these results indicated that TAM-targeted delivery of miR-99b or miR-125a impeded tumor growth by regulating the immune microenvironment.

### Delivery of miR-99b or miR-125a into TAMs of HCC promoted M2-like to M1-like switch of TAMs

**Figure 2:**
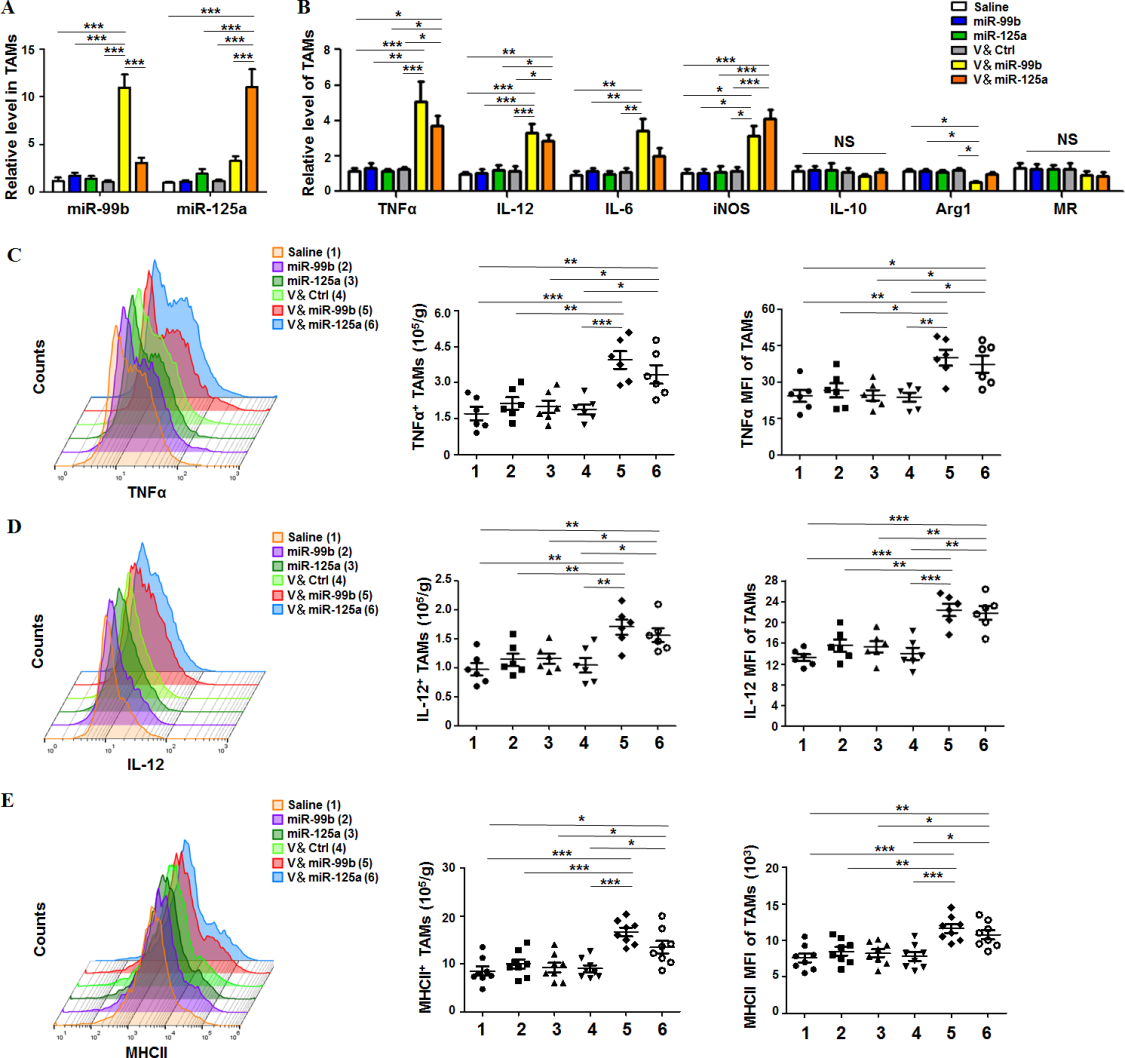
Delivery of miR-99b or miR-125a to TAMs re-educated TAMs toward M1-like phenotype in HCC. (A) TAMs (CD45^+^Ly6G^−^CD11b^+^F4/80^+^) were sorted from orthotopic HCC tumors after different drug treatment and the miR-99b and miR-125a levels were detected by qRT-PCR with U6 as Ctrl (n=4). (B) The mRNA levels of macrophage polarisation-related genes in sorted TAMs were examined by qRT-PCR with β-actin as internal Ctrl (n=4). (C) The cell number of TNFα^+^ TAMs in tumor and the mean fluorescence intensity of TNFα in TAMs were quantitatively compared by FACS (n=6). (D) The cell number of IL-12^+^ TAMs in tumor and the mean fluorescence intensity of IL-12 in TAMs were quantitatively compared by FACS (n=6). (E) The cell number of MHC-II^+^ TAMs in tumor and the mean fluorescence intensity of MHCII in TAMs were quantitatively compared by FACS (n=8). Data are shown as mean±SEM. *p<0.05; **p<0.01; ***p<0.001 by one-way ANOVA with Tukey's multiple comparison test. ANOVA, analysis of variance; HCC, hepatocellular carcinoma; TAM, tumor-associated macrophage.

Next, we analyzed whether TAM-targeted delivery of miR-99b or miR-125a redirected the polarization of TAMs in HCC-bearing mice. After different drug treatments, TAMs and non-TAM cells were sorted (online supplementary figure S8A), and then the levels of miR-99b and miR-125a were detected by qRT-PCR. The result showed that miR-99b or miR-125 was mostly enriched in TAMs but not in non-TAM cells (figure 2A and online supplementary figure S8B). Furthermore, V&miR-99b or V&miR-125 treatment promoted the expression of M1 markers including TNFα, IL-12, IL-6 and iNOS, and decreased the level of Arg1, which is a specific marker of M2 macrophages (figure 2B). We confirmed the phenotype and function of M1-like TAMs by showing that the cell number of TNFα^+^, IL-12^+^ and MHCII^+^ M1-like TAMs and their corresponding mean fluorescence intensity (MFI) increased significantly in V&miR-99b-treated or V&miR-125a-treated mice, especially in V&miR-99b-treated mice (figure 2C–E). Collectively, these results demonstrated that TAM-targeted delivery of miR-99b or miR-125a inhibited tumor growth through reprogramming TAM polarization.

### MiR-99b promoted M1 and suppressed M2 macrophage polarization

**Figure 3:**
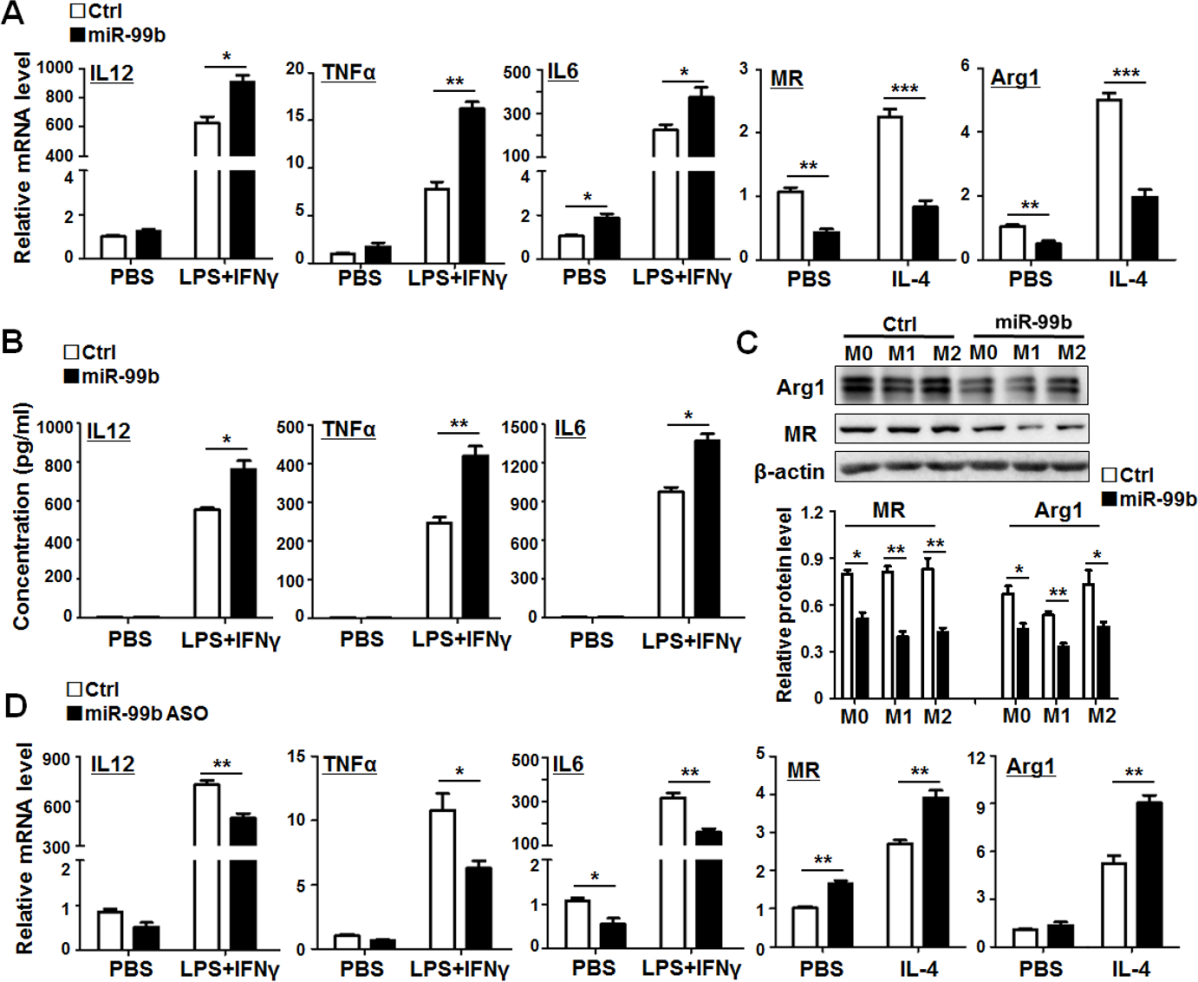
miR-99b promoted M1 and suppressed M2 macrophage polarization. (A) BMDMs were transfected with miR-99b mimics or Ctrl and stimulated with PBS, LPS+IFNγ or IL-4 for 24 hours. The expressions of IL-12, TNFα, IL-6, MR and Arg1 were determined by qRT-PCR (n=3). (B) BMDMs were treated as (A) and the protein levels of IL-12, TNFα and IL-6 in cultured supernatant were measured by ELISA (n=5). (C) BMDMs were treated as (A) and the cells were collected and lysed. The expression of MR and Arg1 in cell lysates was examined by western blot (n=3). (D) BMDMs were transfected with miR-99b antisense oligonucleotides or Ctrl and treated as (A). The mRNA levels of IL-12, TNFα, IL-6, MR and Arg1 were determined by qRT-PCR (n=3). Data are shown as mean±SEM. *p<0.05; **p<0.01; ***p<0.001 by unpaired student’s t-test. BMDMs, bone marrow-derived macrophages.

Based on our findings with miR-125a,^7^ here, we investigated the regulation mechanism of miR-99b on macrophage polarization and function. The results of qRT-PCR, ELISA and western blot indicated that miR-99b promoted the expression of M1 markers including IL-12, TNFα and IL-6, while suppressing the expression of M2 markers, such as MR and Arg1 (figure 3A–C). Consistent with these findings, transfection of miR-99b ASO into BMDMs downregulated M1 markers and upregulated M2 markers compared with those in the control (Ctrl) (figure 3D). These results indicated that miR-99b could promote M1 and suppress M2 polarization in vitro.

**Figure 4:**
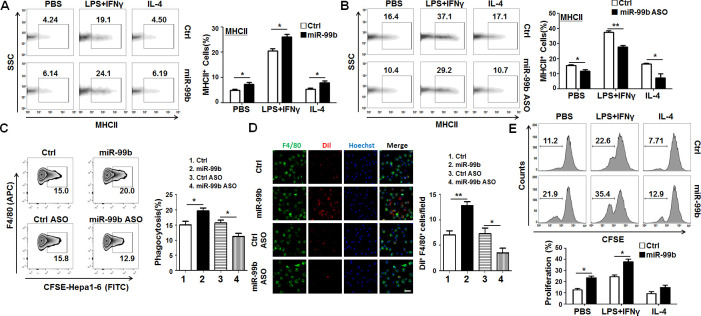
miR-99b enhanced phagocytosis and antigen presentation of macrophages. (A). The MHCII expression in different polarized BMDMs that were transfected with miR-99b mimics or Ctrl was analyzed by FACS (n=3). (B) The MHCII expression in different polarized BMDMs that were transfected with miR-99b ASO or Ctrl was analyzed by FACS (n=3). (C) Phagocytosis of CFSE-labeled Hepa1-6 cells by BMDMs that were transfected with miR-99b mimics or Ctrl, as well as miR-99b ASO or Ctrl, was analyzed by FACS (n=3). (D) BMDMs transfected with miR-99b mimics or Ctrl, as well as miR-99b ASO or Ctrl, were cocultured with Dil-labeled Hepa1-6 cells for 6 hours and the phagocytosis activity of macrophages was observed under a confocal microscope. The number of tumor cells engulfed by macrophages were counted and compared (n=4). (E) Different polarized BMDMs transfected with miR-99b mimics or Ctrl were irradiated and cocultured with CFSE-labeled allogeneic T cells for 24 hours. The T-cell proliferation was determined by FACS (n=4). Data are shown as mean±SEM. *p<0.05; **p<0.01 by unpaired student’s t-test. ASO, antisense oligonucleotides; BMDMs, bone marrow-derived macrophages; CFSE, carboxyfluorescein diacetate succinimidyl ester.

As MHCII expression in macrophages is related to their antigen presentation capability, MHCII has been viewed as a functional marker of M1 macrophages.^28 29^ Next, we analyzed the expression of MHCII in different polarized macrophages by FACS. The results showed that overexpression of miR-99b in BMDMs enhanced MHCII expression in different polarized macrophages, especially in M1 polarized macrophages. Conversely, transfection of miR-99b ASO reduced MHCII expression in different polarized macrophages (figure 4A, B). Moreover, it was revealed that overexpression of miR-99b in natural killer cells increased the ability of Fcγ-mediated phagocytosis, antigen processing and presentation as compared with the Ctrl (online supplementary figure S9A‒D). Similarly, BMDMs transfected with miR-99b exhibited stronger phagocytosis of Hepa1-6 cells, while inhibition of miR-99b could attenuate the phagocytic activity of BMDMs as compared with the Ctrl (figure 4C, D and online supplementary figure S9E). Meanwhile, BMDMs transfected with miR-99b resulted in stronger T-cell proliferation as shown by mixed lymphocyte reaction assay (figure 4E). Taken together, these results suggested that overexpressed miR-99b in macrophages could induce M1 macrophage polarization with enhanced capability of phagocytosis and antigen presentation.

### miR-99b promoted M1 and suppressed M2 polarisation by targeting κB-Ras2 and/or mTOR

**Figure 5:**
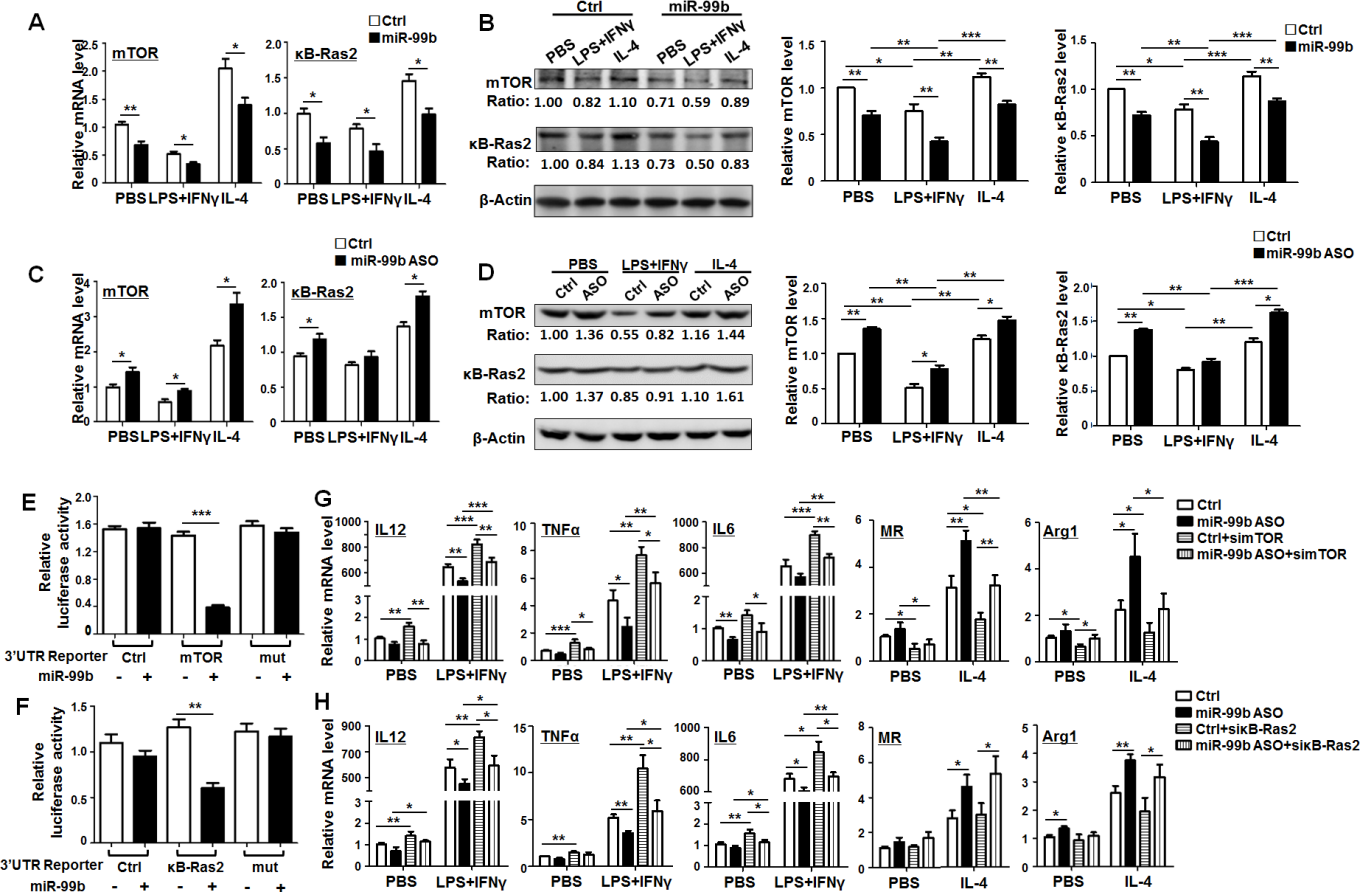
miR-99b promoted M1 and suppressed M2 polarization by targeting κB-Ras2 and/or mTOR. (A) BMDMs transfected with miR-99b mimics or Ctrl were treated with PBS, LPS+IFNγ or IL-4. The mRNA levels of mTOR (left) and κB-Ras2 (right) were determined by qRT-PCR (n=4). (B) BMDMs transfected with miR-99b mimics or Ctrl were treated as (A). The protein levels of mTOR and κB-Ras2 were examined by western blot and quantified (n=4). (C) BMDMs transfected with miR-99b ASO or Ctrl were treated as (A), the expression of mTOR (left) and κB-Ras2 (right) was determined by qRT-PCR (n=4). (D) BMDMs transfected with miR-99b ASO or Ctrl were treated as (A). The protein levels of mTOR and κB-Ras2 were examined by western blot and quantified (n=4). (E, F) HEK293 cells were transfected with miR-99b mimics or Ctrl, as well as reporter plasmid containing wild-type or mutant 3′-UTRs of mTOR (E) or κB-Ras2 (F). Luciferase activity was detected 24 hours after the transfection (n=4). (G, H) BMDMs transfected with miR-99b ASO or Ctrl, as well as simTOR (G) or siκB-Ras2 (H) was treated with PBS, LPS+IFNγ or IL-4. The mRNA levels of indicated genes were measured by qRT-PCR (n=3). Data are shown as mean±SEM. *p<0.05; **p<0.01; ***p<0.001 by unpaired student’s t-test. ASO, antisense oligonucleotides; BMDMs, bone marrow-derived macrophages.

Next, we predicted the target genes of miR-99b with five miRNA target gene prediction databases including DIANA, MIRDB, Pictar, RNA22 and Targetscan. Then, we screened out mTOR and κB-Ras2 as potential targets of miR-99b using the Venn diagram. The 3′-UTR of mTOR and κB-Ras2 recognized by miR-99b was shown in online supplementary figure S10A. BMDMs transfected with miR-99b mimics, miR-99b ASO or Ctrl were treated with PBS, LPS+IFNγ or IL-4 for 24 hours and the levels of mTOR and κB-Ras2 were determined by qRT-PCR and western blot. The results showed that mTOR and κB-Ras2 expression was significantly decreased in BMDMs transfected with miR-99b (figure 5A, B). This effect was completely reversed after miR-99b ASO transfection (figure 5C, D). Moreover, reporter assay showed that miR-99b suppressed luciferase activity of cells transfected with reporter plasmids containing the wild-type 3′-UTR of mTOR or κB-Ras2, whereas disruption of the proximal seed sequence (289–295 bp) in mTOR 3′-UTR or the seed sequence (733–738 bp) in κB-Ras2 3′-UTR abrogated this effect (figure 5E, F). Taken together, these data suggested that miR-99b downregulated the expression of mTOR and κB-Ras2 via their 3′-UTRs in macrophages.

To further investigate the contribution of mTOR and κB-Ras2 to miR-99-regulated macrophage polarization, we designed three siRNA against mTOR or κB-Ras2 and then determined their knockdown efficiency by qRT-PCR (online supplementary figure S10B). Knockdown of mTOR in BMDMs enhanced M1 and decreased M2 polarization. However, knockdown of κB-Ras2 only resulted in upregulation of M1 related markers but had no significant influence on M2-related markers (online supplementary figure S10C). Moreover, transfection of miR-99b ASO into BMDMs downregulated the expression of M1 markers and upregulated the expression of M2 markers, while mTOR knockdown reversed the effect of miR-99b ASO (figure 5G). In contrast, knockdown of κB-Ras2 only rescued the expression of M1 markers that were downregulated by miR-99b ASO (figure 5H). As expected, knockdown of mTOR or κB-Ras2 in miR-99b ASO-overexpressing macrophages could recover the ability of T-cell proliferation (online supplementary figure S10D). Moreover, TAM-targeted delivery of simTOR or siκB-Ras2 inhibited miR-99b antagomir-triggered tumor growth by promoting M1 polarization and antigen presentation (online supplementary figure S11). Taken together, these results indicated that miR-99b promoted M1 and suppressed M2 polarization by targeting κB-Ras2 and/or mTOR.

### miR-99b promoted M1 macrophage polarization by enhancing NF-κB signaling through inhibiting the expression of mTOR and κB-Ras2

**Figure 6:**
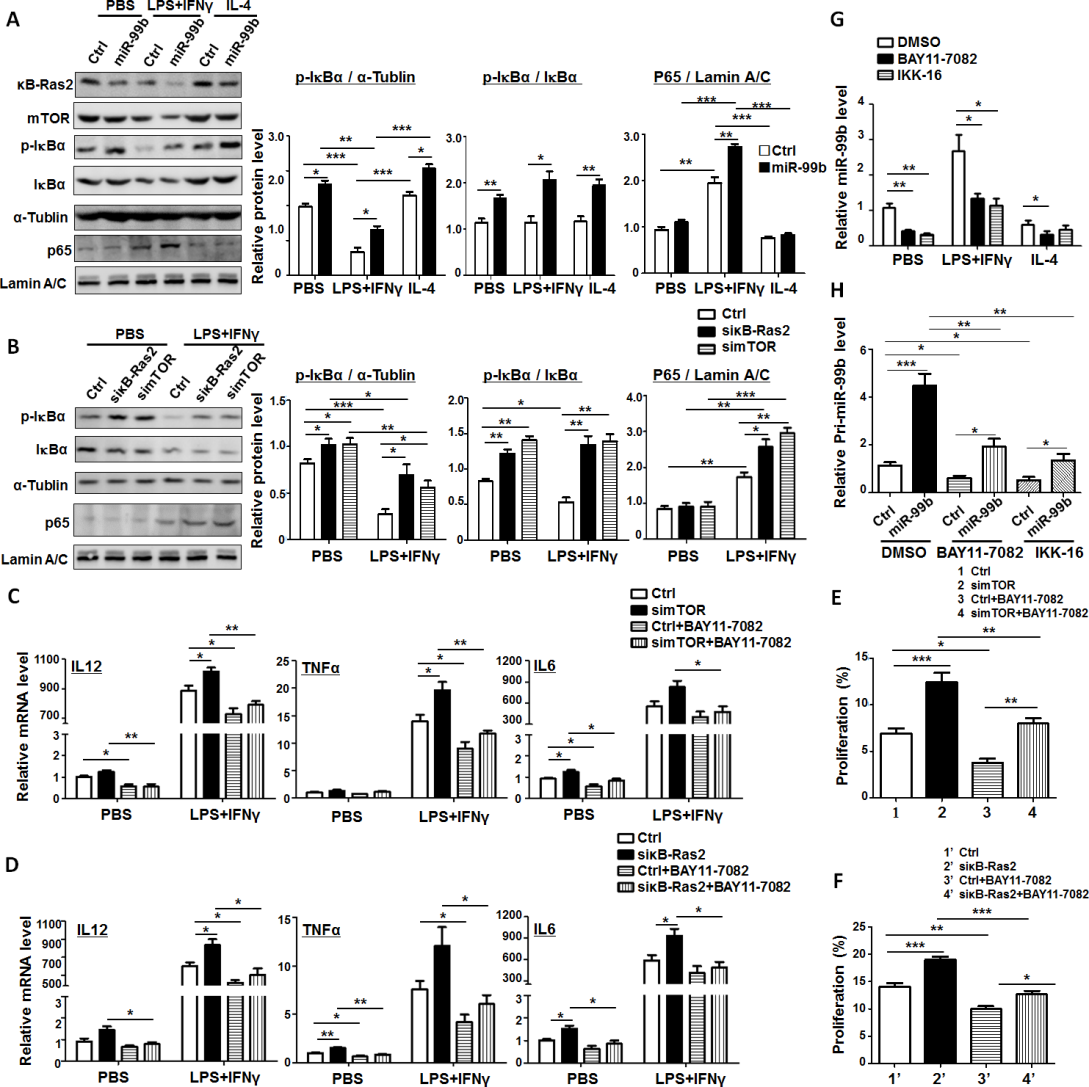
The miR-99b/NF-κB positive feedback loop enhanced M1 macrophage polarization by inhibiting mTOR and κB-Ras2. (A) RAW264.7 cells transfected with miR-99b mimics or Ctrl were treated with PBS, LPS+IFNγ or IL-4 for 24 hours. The protein levels of indicated genes were detected by western blot and quantitatively compared (n=3). (B) RAW264.7 cells transfected with simTOR, siκB-Ras2 or Ctrl was treated with PBS or LPS+IFNγ for 24 hours, respectively. The protein levels of indicated genes were detected by western blot. The relative protein levels of indicated genes were quantitatively compared (n=3). (C) BMDMs transfected with simTOR or Ctrl were treated with DMSO or BAY11-7082 for 12 hours, and stimulated with PBS, LPS+IFNγ or IL-4 for 24 hours. The expression of indicated genes was determined by qRT-PCR (n=4). (D) BMDMs transfected with siκB-Ras2 or Ctrl were treated as (C). The expression of indicated genes was determined by qRT-PCR (n=4). (E, F) BMDMs were treated as (C) or (D), and then irradiated and cocultured with CFSE-labeled allogeneic T cells for 24 hours. The proliferation of T cells was determined by FACS (n=4). (G) BMDMs were treated with DMSO, BAY11-7082 or IKK-16 for 12 hours and then stimulated with PBS, LPS+IFNγ or IL-4 for 24 hours. The expression of miR-99b was detected by qRT-PCR with U6 RNA as Ctrl (n=3). (H) BMDMs were treated with DMSO, BAY11-7082 or IKK-16 for 12 hours, and then were transfected with miR-99b mimics or Ctrl. The expression of primary miR-99b (pri-miR-99b) was detected by qRT-PCR 24 hours post-transfection (n=4). Data are shown as mean±SE.M. *p<0.05; **p<0.01; ***p<0.001 by unpaired student’s t-test (A–D, G and H) or one-way ANOVA with Tukey's multiple comparison tests (E and F). ANOVA, analysis of variance; BMDMs, bone marrow-derived macrophages; CFSE, carboxyfluorescein diacetate succinimidyl ester.

NF-κB signaling is a classical signaling pathway for mediating macrophage polarization. M1 stimuli, such as TLR ligands, TNFα and IL-1β, induce macrophage activation primarily by activating NF-κB signaling.^30^ Thus, we wondered whether miR-99b promoted M1 polarization by enhancing NF-κB signaling through downregulating mTOR and κB-Ras2. Western blot showed that transfection with miR-99b in macrophages followed with LPS+IFNγ stimulation could trigger the phosphorylation and degradation of IκBα leading to the translocation of p65 into the nucleus, and the expression of κB-Ras2 and mTOR was decreased, indicating that miR-99b overexpression in macrophages promoted the activation of NF-κB signaling (figure 6A). Consistently, knockdown of κB-Ras2 or mTOR in BMDMs followed with LPS+IFNγ stimulation induced the same phenomenon as miR-99b overexpressed macrophages (figure 6B). Meanwhile, administration of the NF-κB inhibitor, BAY11-7082, into BMDMs impeded the upregulation of M1 markers, such as IL-12, TNFα and IL-6, which was induced by knockdown of mTOR or κB-Ras2 (figure 6C, D). Moreover, T-cell proliferation was reduced significantly in mTOR or κB-Ras2 knockdown macrophages with blockade of NF-κB signaling (figure 6E, F). These results demonstrated that the axis of miR-99b/mTOR or miR-99b/κB-Ras2 regulated M1 macrophage activation and function through NF-κB signaling. More interestingly, BMDMs were treated with BAY11-7082, IKK-16 and DMSO followed by different polarized stimulation, the expression of miR-99b after NF-κB signaling blockade was significantly repressed compared with that in the Ctrl. Overexpressed miR-99b in this system could partially rescue the effect of NF-κB signaling blockade, indicating that the positive feedback of regulation between miR-99b and NF-κB signaling could enhance M1 macrophage polarization by inhibiting the expression of mTOR and κB-Ras2 (figure 6G, H).

### miR-99b attenuated M2 polarization by repressing the mTOR/IRF4 axis in macrophages

**Figure 7:**
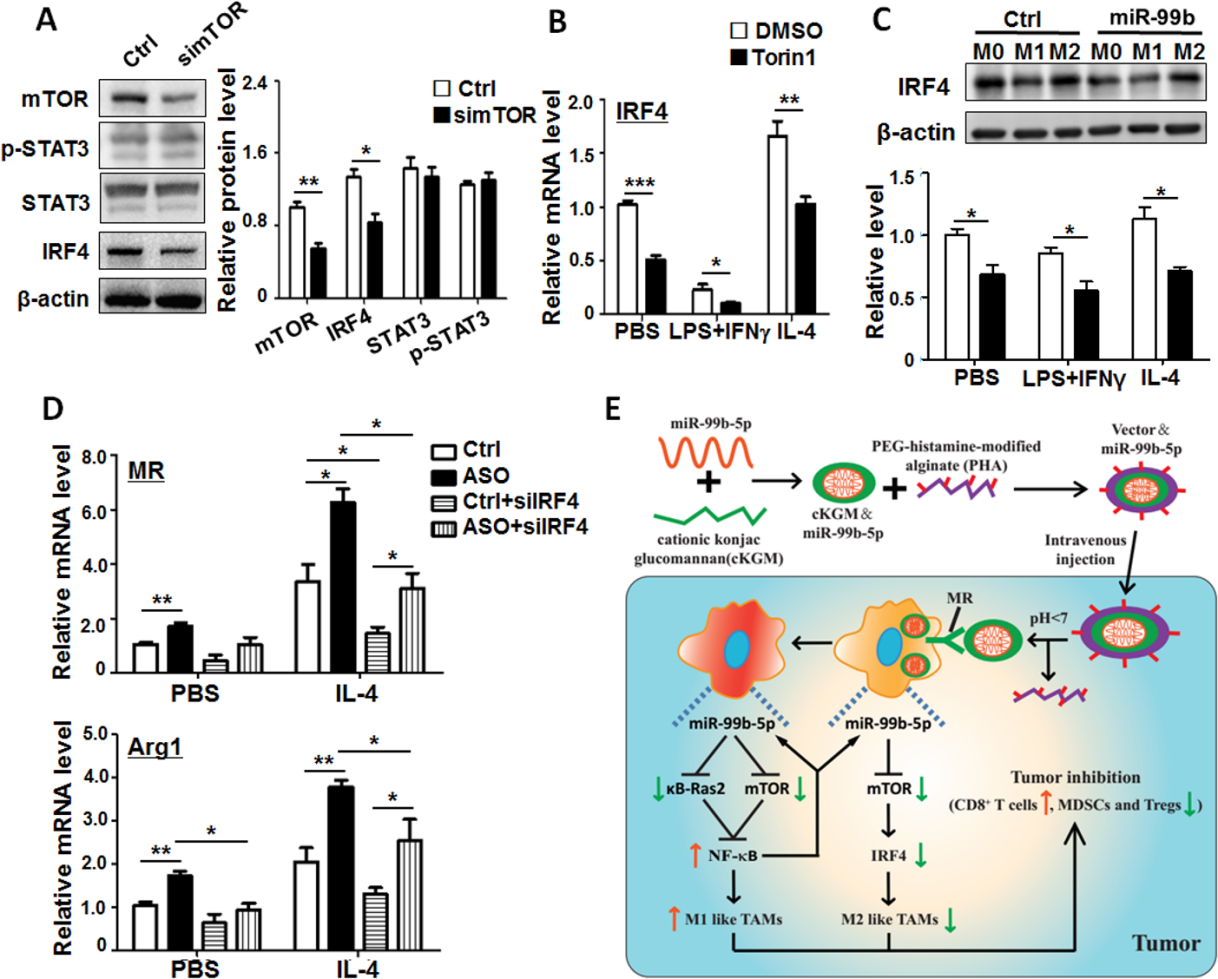
MiR-99b attenuated M2 polarization via repressing the mTOR/IRF4 axis in macrophages. (A) Cell lysates from BMDMs transfected with simTOR or Ctrl were analyzed by western blot. The relative protein levels of mTOR, STAT3, p-STAT3 and IRF4 were quantitatively compared (n=3). (B) BMDMs were pretreated with DMSO or Torin1 for 6 hours, and then stimulated with PBS, LPS+IFNγ or IL-4. The IRF4 expression was examined by qRT-PCR (n=4). (C) BMDMs transfected with miR-99b mimics or Ctrl were stimulated with PBS, LPS+IFNγ or IL-4 for 24 hours. The relative protein levels of IRF4 were quantitatively compared by western blot (n=3). (D) BMDMs were transfected with miR-99b ASO or Ctrl and siIRF4 followed by PBS or IL-4 stimulation for 24 hours. The expression of indicated genes was determined by qRT-PCR (n=4). (E) Schematic diagram of TAM-targeted delivery of miR-99b inhibiting tumor growth by reprogramming TAM phenotype. Data are shown as mean±SEM. *p<0.05; **p<0.01; ***p<0.001 by unpaired student’s t-testonline supplementary additional file. BMDMs, bone marrow-derived macrophages.

Many studies have demonstrated that mTOR can regulate M2 macrophage polarization via transcription factors STAT3 and IRF4.^31 32^ Our western blot results showed that knockdown of mTOR in BMDMs resulted in the reduction of mTOR and IRF4 but had no significant influence on STAT3 and p-STAT3 expression (figure 7A). In addition, blockade of mTOR signaling in BMDMs by Torin1, an inhibitor of mTOR-mediated signaling, significantly decreased IRF4 expression (figure 7B). To further investigate whether IRF4 was a key downstream molecule of miR-99b-driven M2 macrophage polarization, we monitored IRF4 expression in different polarized BMDMs after miR-99b transfection. The results showed that miR-99b repressed the expression of IRF4 in different polarized BMDMs compared with the Ctrl (figure 7C). Furthermore, miR-99b ASO and/or siIRF4 were transfected into BMDMs and M2 polarization was induced with IL-4 stimulation. The results showed that miR-99b ASO promoted M2 polarization by increasing M2 marker expression, while knockdown of IRF4 hindered the effect (figure 7D and online supplementary figure S10E). These results indicated that miR-99b attenuated M2 polarization by repressing the mTOR/IRF4 axis in macrophages.

Finally, in order to confirm the axis of miR-99b-mTOR and/or κB-ras2 and miR-99b-mTOR/IRF4 in vivo, we detected the expression of κB-ras2, mTOR and IRF4 in TAMs sorted from tumor-bearing mice after delivery of saline, miR-99b, V＆Ctrl and V＆miR-99b. As shown in online supplementary figure S12A, V＆miR-99b treatment could significantly inhibit the expression of mTOR, κB-ras2 and IRF4 in TAMs compared with saline, miR-99b or V＆Ctrl treatment. More importantly, we found that the expression of mTOR and κB-Ras2 was higher in sorted TAMs from tumor tissues than in adjacent tissues (online supplementary figure S12B), which was corresponded to miR-99b expression in liver cancer patients, indicating that miR-99b might reprogram the TAM phenotype by regulating mTOR and/or κB-ras2 expression during tumorigenesis, and that TAM-targeted miR-99b delivery could be a potential therapy strategy for cancer in the future.

In summary, TAM-targeted delivery of miR-99b inhibited tumor growth by reprograming the TAM phenotype from protumor to antitumor. On the one hand, miR-99b overexpression in TAMs reduced the M2-like TAM phenotype by repressing mTOR/IRF4 expression. On the other hand, overexpression of miR-99b in TAMs promoted the switching of M2-like to M1-like TAMs by enhancing NF-κB activity through suppressing the expression of κB-Ras2 and mTOR. Activated NF-κB further promoted miR-99b expression by a positive feedback loop. Finally, the amplified M1-like effect in miR-99b-overexpressed TAMs resulted in tumor regression by reprograming the antitumor immune microenvironment, such as increased CD8^+^T cells and decreased MDSCs and Tregs (figure 7E).

## Discussion

Several studies have reported that miR-99b can regulate myeloid cell differentiation and macrophage activation.^13 33^ Recently, Huber *et al* have unveiled that melanoma extracellular vesicles containing a set of miRNAs, such as miR-99b, miR-146a, miR-155 and miR-125a, can enhance the conversion of monocytes into monocytic MDSCs, which results in immunotherapy resistance in melanoma patients.^14^ In the current study, we found that miR-99b overexpression promoted the differentiation of CD11b^+^ monocytes into macrophages rather than granulocytes under GM-CSF stimulation (online supplementary figure S2). Moreover, the overexpression of miR-99b in macrophages promoted M1 polarization by targeting κB-Ras2 and mTOR while inhibiting M2 polarization via mTOR/IRF4 targeting (figure 7D). This regulatory axis also existed in human and murine TAMs. To the best of our knowledge, this study is the first to explore the role of miR-99b in macrophage polarization and function. Previously, our work has shown that miR-125a participates in M1 macrophage polarization,^7^ because miR-99b and miR-125a belong to one miRNA cluster that often coordinate their role in cell differentiation,^12 13^ we thus proposed that miR-99b and miR-125a could regulate macrophage M1 polarization in a cluster way. However, unexpectedly, we found that the capacity of miR-99b-mediated M1 macrophage polarization and function was almost equal to that of the miR-99b-miR-125a cluster, and even more than miR-125a (data not shown), indicating that miR-99b might be a key player in regulating M1 macrophage polarization and function.

Here, we found that miR-99b not only promoted macrophage M1 polarization but also enhanced its capability of phagocytosis and antigen presentation, which could be associated with stronger antitumor ability after being delivered into TAMs. Multiple signaling pathways have been reported to involve in macrophage phagocytosis and antigen presentation including NF-κB and mTOR signaling. Wong *et al* demonstrate that during bacterial clearance by macrophage, NF-κB signaling is activated by lysosomal degradation to maintain continuous phagocytosis of bacteria.^34^ Moreover, other studies have confirmed the important role of NF-κB signaling in antigen presentation by exploring the function of several kinases acting upstream of NF-κB.^35–37^ mTOR signaling has also been reported to participate in regulating phagocytosis and antigen presentation. Given that tubular lysosomes are required for phagosome maturation and antigen presentation, Saric *et al* demonstrate that mTOR is responsible for LPS-induced lysosome tubulation and MHCII expression in macrophages via augmented membrane-associated Arl8b expression, a lysosomal GTPase that can promote lysosome trafficking in a kinesin-dependent manner.^38^ Interestingly, in our study, NF-κB and mTOR were verified as downstream molecules of miR-99b during macrophage polarization, therein mTOR was a direct target of miR-99b. However, further experiments are needed to clarify whether NF-κB or mTOR signaling is involved in miR-99b-mediated macrophage phagocytosis and antigen presentation and its underlying regulation mechanism.

The switch of protumor M2-like to antitumor M1-like TAMs has been viewed as a promising anticancer therapy.^15^ When miR-99b and/or miR-125a were delivered into TAMs using nanoparticles, we found that both tumor sizes in orthotopic inoculated HCC-bearing and subcutaneous transplanted LLC-bearing mice were impeded significantly. Further mechanistic studies showed that miR-99b or miR-125a delivery reduced tumor growth by repolarising M2-like TAMs to M1-like TAMs followed by immunosuppressive microenvironment abrogation (figure 7D and ref.^7^). Although several TAM-targeting agents, such as CCR2 inhibitors, anti-CSF1R antibodies and anti-CD40 agonists, have been applied in clinical trials owing to their roles in blocking macrophage recruitment, survival and eliminating immunosuppression,^2 6 15^ some drawbacks including off-targets and side effects on their application to cancer therapy, should be given more attention. For example, Bonapace *et al* report that cessation of anti-CCL2 treatment might accelerate the death of tumor-bearing mice by rebounding monocyte recruitment and enhancing tumor angiogenesis and metastasis.^39^ Similarly, interruption of CSF-1R blockade can also cause monocyte-derived macrophage accumulation leading to tumor recurrence.^40^ In addition, depletion of TAMs by systemic delivery of clodronate-encapsulated liposomes can suppress tumor growth by inducing macrophage apoptosis.^41^ However, some studies imply that this systemic depletion of macrophages may exacerbate tumor progression due to the indiscriminate clearance of antitumor CD169^+^ macrophages.^42^ Because of these limitations of TAM-centered immunotherapy, it is urgent to develop more precise and specific TAM-targeted strategies for cancer treatment. Meanwhile, owing to the potential clinical application of miRNA delivery, our study suggested that miR-99b might be an ideal drug candidate for tumor therapy by targeting TAMs.

Polymeric nanoparticles have been adapted for drug delivery to cancer and macrophages based on their response to the acidic TME. Recently, Wang *et al* has developed microenvironment-responsive nanoparticles carrying with IL-12 and found that IL-12 is distributed in the TME by nanoparticle delivery and re-educates TAMs toward an antitumor phenotype.^43^ Furthermore, in order to specifically target TAMs rather than other cells, TAM-mediated endocytosis that is triggered by ligand-receptor interaction becomes a new target option.^44^ Among them, the mannose receptor (MR) is highly expressed on the surface of M2-like TAMs that can efficiently induce internalization. In view of this, Huang *et al* design one nanoparticle that possesses an affinity for MR on TAMs and can respond to the low pH in TME. Taking advantage of this delivery system, TAM-targeted delivery of let-7b or miR-99b (our study) leads to tumor growth regression by reprogramming TAM function and reversing the immunosuppressive microenvironment.^24^ However, MR is also expressed by TIDCs, indicating that targeting specific macrophage is still a major challenge. As such, it has become urgent to advance our understanding of the function, origin and diversity of macrophages in order to develop more precise cancer therapy strategies by targeting TAMs.

## Conclusions

In summary, TAM-targeted delivery of miR-99b inhibited tumor growth by reprograming the TAM phenotype from protumor to antitumor. On the one hand, miR-99b overexpression in TAMs reduced the M2-like TAM phenotype by repressing mTOR/IRF4 expression. On the other hand, overexpression of miR-99b in TAMs promoted M2-like to switch to M1-like TAMs by enhancing the activity of NF-κB through suppressing κB-Ras2 and mTOR expression. Activated NF-κB in turn promoted the miR-99b expression by a positive feedback loop. The miR-99b/mTOR and miR-99b/κB-Ras2 axis was also verified in TAMs of tumor-bearing mice and patients with live cancer. Finally, the amplified M1-like effect in miR-99b overexpressed TAMs resulted in tumor regression by reprogramming the antitumor immune microenvironment, such as increased CD8^+^T cells and decreased MDSCs and Treg cells (figure 7E).
