# Supplementary material for: Targeted delivery of miR-99b reprograms tumor-associated macrophage phenotype leading to tumor regression
Source: J Immunother Cancer. 2020 Sep 18;8(2):e000517. doi: 10.1136/jitc-2019-000517 (PMC7511616; doi:10.1136/jitc-2019-000517)
Supplement: Supplementary data [file jitc-2019-000517supp001.pdf]

Additional file 1

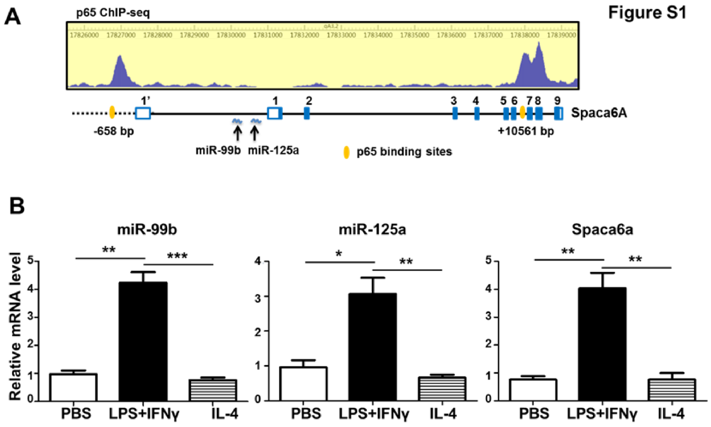

Figure S1. miR-99b and miR-125a simultaneous transcription under LPS + IFNγ stimulation. A. Schematic diagram of *Spaca6A* with miR-99b/miR-125a cluster and two binding sites of p65 (in yellow). B. miR-99b, miR-125a and Spaca6a were transcribed in BMDMs after LPS + IFNγ stimulation. Data are shown as mean ± S.E.M. *, $P < 0.05$; **, $P < 0.01$; ***, $P < 0.001$ by unpaired student t-test.

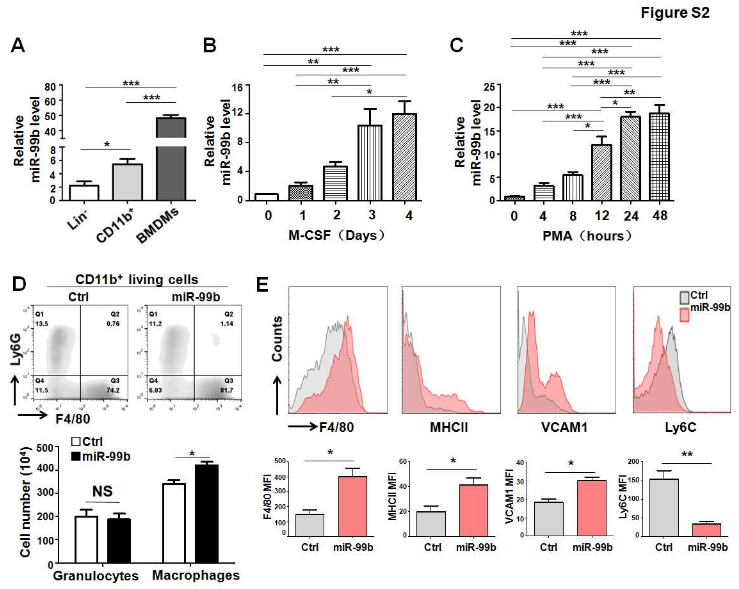

Figure S2. miR-99b was involved in myeloid cell differentiation. A. lineage$^-$(Lin$^-$) bone marrow (BM) and CD11b$^+$ monocytes were sorted by MACS. Meanwhile, monocytes were cultured in the presence of M-CSF for 7 days to obtain BM-derived macrophages (BMDMs). The expression of miR-99b in different cells was detected by qRT-PCR (n = 3). B. The expression of miR-99b during monocytes differentiation into macrophages was detected by qRT-PCR (n=3). C. THP1 monocytes were induced to differentiate into macrophages with PMA treatment, and the expression of miR-99b were detected at different time points by qRT-PCR (n=3). D. BM transfected with miR-99b mimics or control oligonucleotides (Ctrl) were cultured with GM-CSF, and the number of granulocytes (CD11b$^+$Ly6G$^+$F4/80$^-$) and macrophages (CD11b$^+$Ly6G$^-$F4/80$^+$) were determined by FACS (n=3). E. CD11b$^+$ monocytes transfected with miR-99b mimics or control oligonucleotides (Ctrl) were induced to differentiate into macrophages in the presence of M-CSF for 4 days. The mean fluorescence intensity (MFI) of macrophage maturation-related molecules, such as MHCII, VCAM1 and Ly6C, was analysed and compared by FACS (n=3). Data are shown as mean ± S.E.M. *, $P < 0.05$; **, $P < 0.01$; ***, $P < 0.001$ by unpaired student t-test.

Wang L, *et al. J Immunother Cancer* 2020; 8:e000517. doi: 10.1136/jitc-2019-000517

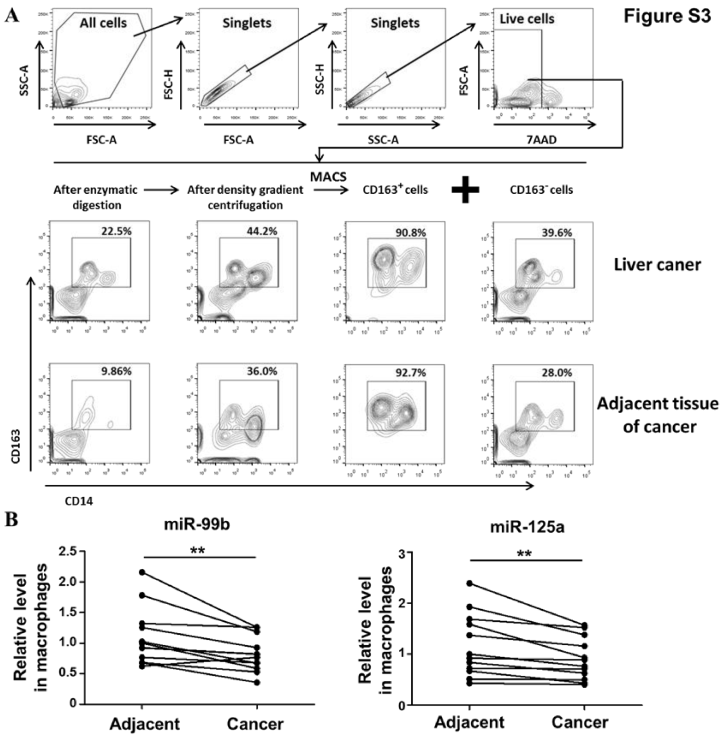

Figure S3. Expression of miR-99b and miR-125a in TAMs sorted from tumour and adjacent tissues in patients with liver cancer. A. The $CD14^{+}CD163^{+}$ TAMs were sorted by MACS following with enzymatic digestion and density gradient centrifugation as previously described [26]. The purity of TAMs was confirmed by FACS. B. The expression of miR-99b or miR-125a in sorted TAMs as shown in (A) were determined by qRT-PCR with U6 as internal control (n=12). Data are shown as mean ± S.E.M. *, $P < 0.05$; **, $P < 0.01$ by paired student t-test.

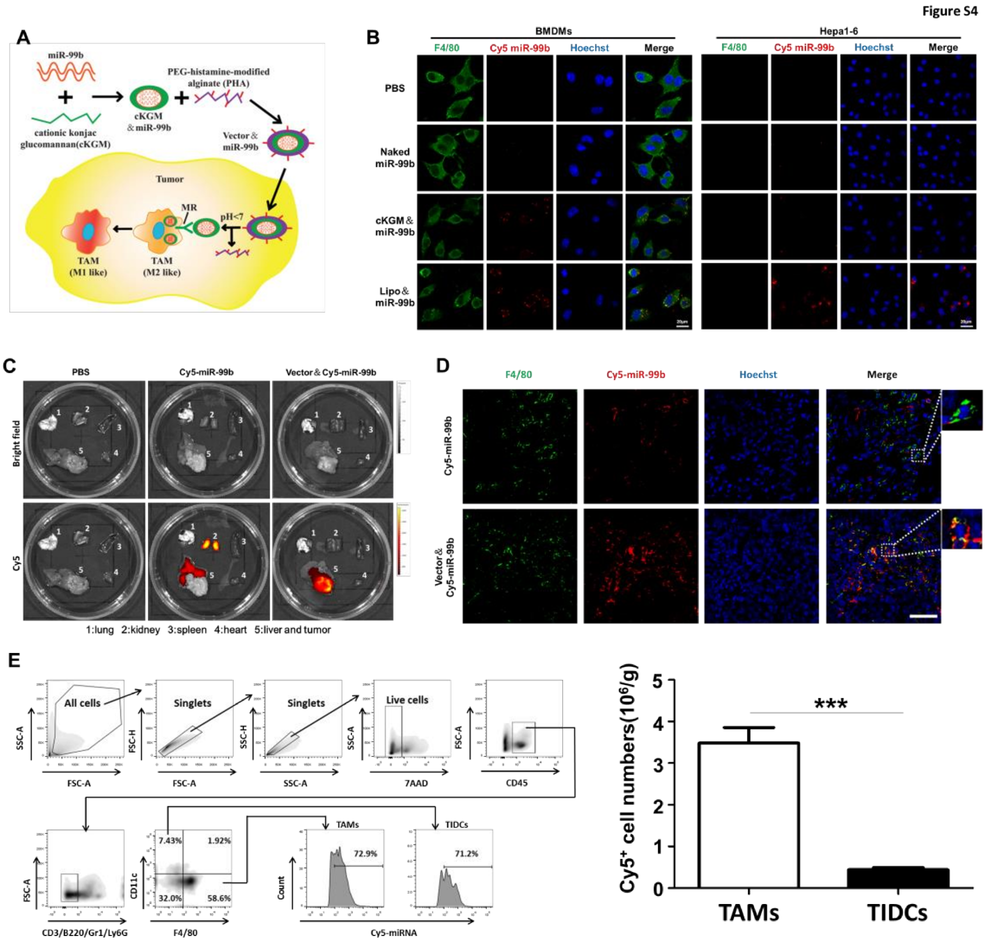

Figure S4. Distribution of miRNA in hepatic carcinoma tissue, TAMs and TIDCs after drug delivery. A. Schematic diagram of TAM-targeted miRNAs (eg. miR-99b) delivery system was shown. B. Cy5-lablled miR-99b was transfected into cultured BMDMs or Hepa1-6 cells. Then, the location of Cy5-miR-99b was observed and photographed by confocal microscopy. Cell nuclei were stained with Hoechst. C. The distribution of miR-99b in different organs of tumour-bearing mice were observed by IVIS Lumina system at 6 h after PBS, naked Cy5-miR-99b and vector＆Cy5-miR-99b administration via mouse tail vein. D. Tumour sections of tumour-bearing mice were obtained from (C) and stained with FITC-F4/80 antibody for further observation under confocal microscopy. E. The $Cy5^{+}$ cells in TAM and TIDC cell population were detected by FACS and the cell number was calculated and compared (n=3). Data are shown as mean ± S.E.M. *, $P < 0.05$; **, $P < 0.01$; ***, $P < 0.001$ by unpaired student

t-test.

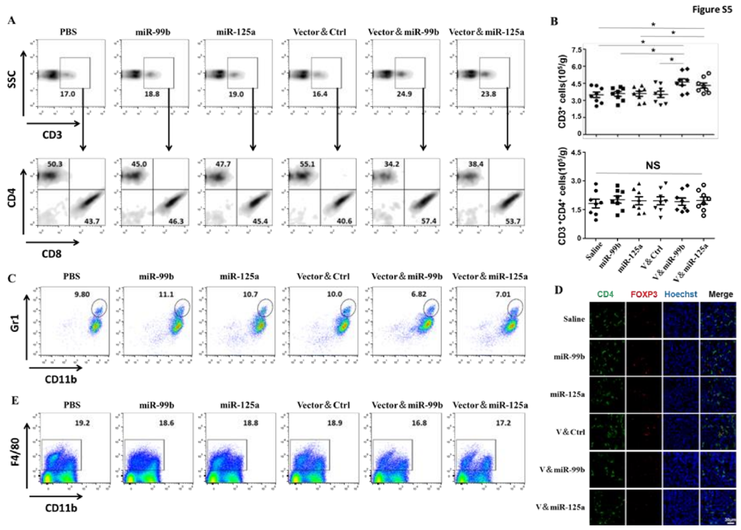

Figure S5. The immune cell phenotype in HCC-bearing mice after different drug treatment. A and B. The cell phenotype of $CD3^{+}CD4^{+}$ and $CD3^{+}CD8^{+}$ T cells was analysed by FACS (A) and the cell number was quantitatively compared (B) (n=8). C. $Gr1^{+}CD11b^{+}$ MDSCs were analysed by FACS. D. The hepatic tumour sections were stained with CD4 and FOXP3 and observed by confocal microscopy. E. $F4/80^{+}CD11b^{+}$ TAMs were analysed by FACS. Data are shown as mean ± S.E.M. *, $P < 0.05$ by one-way ANOVA with Tukey's multiple comparison tests.

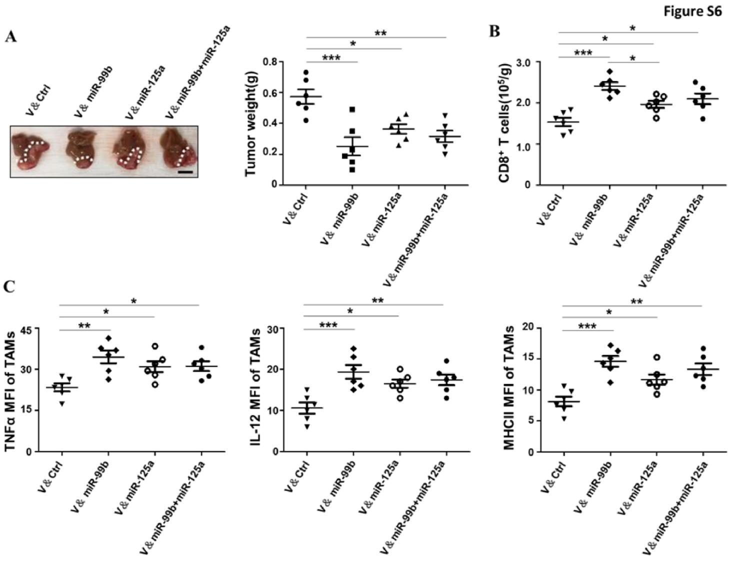

Figure S6. Anti-tumour effect of TAM-targeted delivery miR-99b and/or miR125a in HCC mouse model. A. tumours were isolated from HCC-bearing mice at day 29 after different drug treatments and photographed and weighed (n=6). B. The cell number of $CD8^+$ T cells in tumours was analysed and compared by FACS (n=6). C. The MFI of $TNF\alpha^+$, $IL\text{-}12^+$ and $MHCII^+$ TAMs in HCC-bearing mice was measured by FACS (n=6). Data are shown as mean ± S.E.M. *, P < 0.05; **, P < 0.01; ***, P < 0.001 by one-way ANOVA with Tukey's multiple comparison tests.

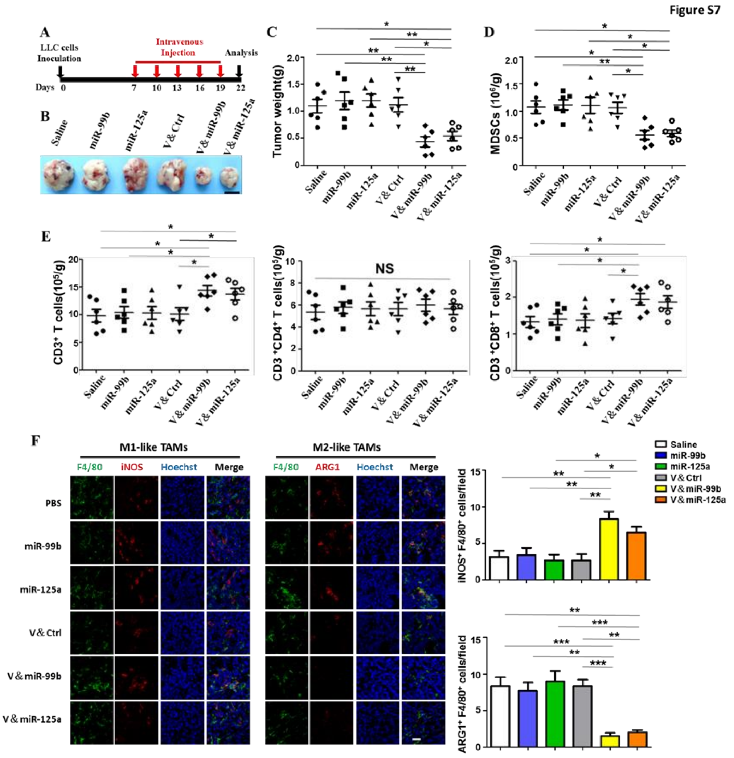

Figure S7. Anti-tumour ability of TAM-targeted delivery of miR-99b or miR125a in subcutaneous LLC mouse model. A. The schedule of TAM-targeted delivery of miR-99b or miR-125a into subcutaneous lung tumour was shown. B. Tumours were isolated from LLC-bearing mice at day 22 after different drug treatments, and then were photographed (n=6). C. Tumour weight was measured as shown in (B). D and E. The cell number of MDSCs (D) and $CD3^{+}$, $CD3^{+}CD4^{+}$ and $CD3^{+}CD8^{+}$ T cells (E) in tumour was analysed by FACS and compared among each group (n=6). F. Lung tumour sections were stained with F4/80, iNOS (M1 marker) or Arg1 (M2 marker) and observed by confocal microscopy. Nuclei were stained with Hoechst. The M1- or M2- TAMs per field were quantitatively compared (n=6). Data are shown as mean ± S.E.M. *, P < 0.05; **, P < 0.01; ***, P < 0.001 by one-way ANOVA with Tukey's multiple comparison tests.

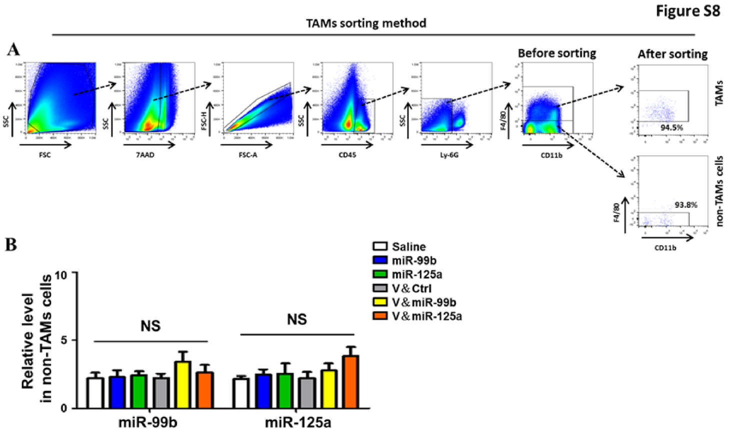

Figure S8. Sorting strategy of TAMs from murine liver tumour. A. The gating strategy of TAMs (7AAD$^-$CD45$^+$Ly6G$^-$CD11b$^{+/lo}$F4/80$^+$) from HHC-bearing mice before sorting. The cell purity was analysed after sorting. B. Non-TAM cells (7AAD$^-$CD45$^+$Ly6G$^-$F4/80$^-$) were sorted as shown in (A), and the expression of miR-99b or miR-125a were determined by qRT-PCR with U6 as internal control (n=4). Data are shown as mean ± S.E.M. One-way ANOVA with Tukey's multiple comparison tests was performed.

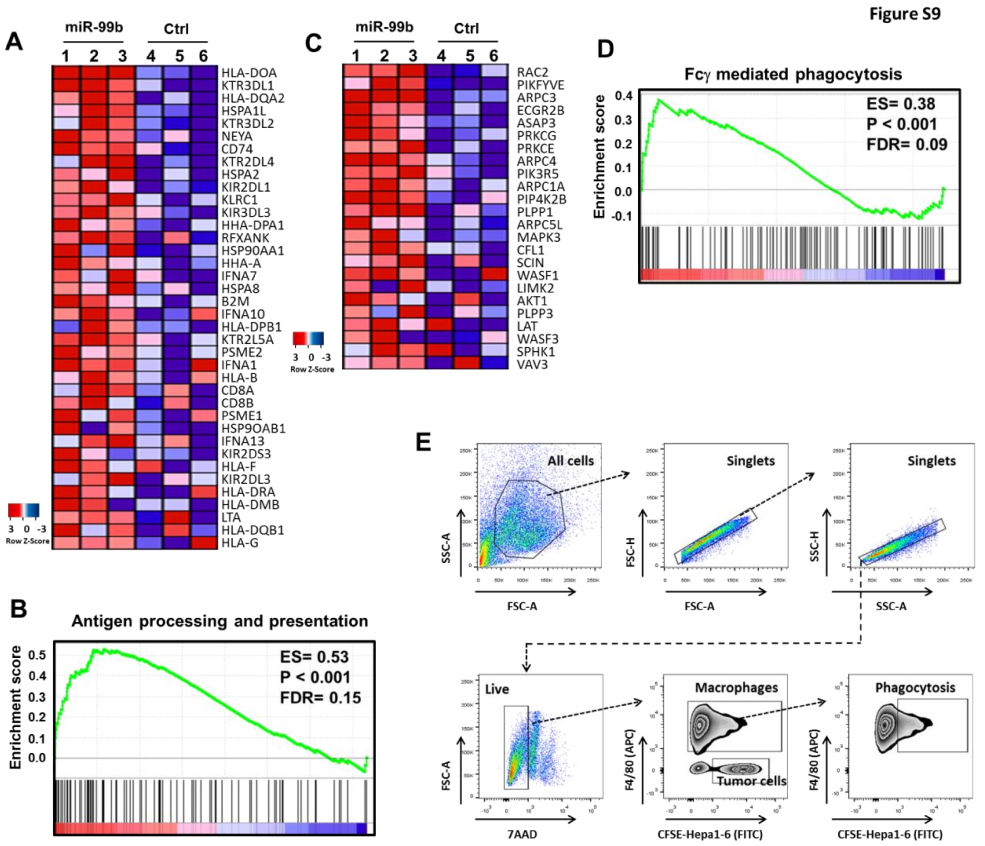

Figure S9. Natural killer (NK) cells transfected with miR-99b exhibited the enhanced phagocytosis, antigen processing and presentation by GSEA. A and B. RNA-sequencing data of human NK cells transfected with miR-99b or control were download and analysed (GSE69555) [12]. The significant genes with the role of antigen processing and presentation (A) and relevant GSEA (B) were shown. C and D. The significant genes with the role of Fcγ-mediated phagocytosis (C) and relevant GSEA (D) were shown. E. The gating strategy of phagocytosis assay was displayed.

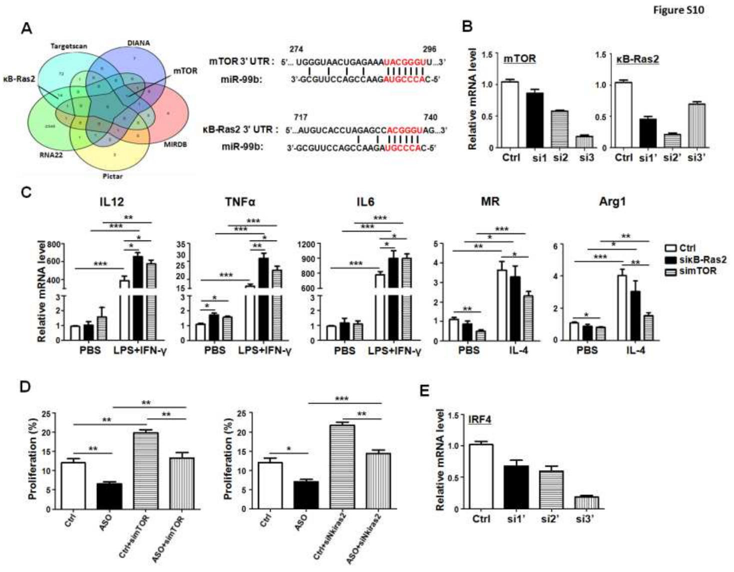

Figure S10. Knockdown of mTOR and κB-Ras2 in macrophages promoted M1 and suppressed M2 polarisation. A. The miR-99b target genes were predicted by five different databases and displayed with Venn diagram (left panel). The recognized sequence of the 3'-UTR of mTOR (nucleotides 289 ~ 295) and κB-Ras2 (nucleotides 733 ~ 738) was aligned with the seed sequence of miR-99b (right panel). B. The efficiency of knockdown mTOR (left) and κB-Ras2 (Right) expression in BMDM via siRNA was determined by qRT-PCR (n=3). C. BMDMs transfected with simTOR, siκB-Ras2, or control siRNA were stimulated with PBS, LPS + IFNγ or IL-4. After 24 h, the mRNA level of indicated genes was determined by qRT-PCR (n = 3). D. BMDMs transfected with miR-99b ASO or control (Ctrl) as well as simTOR or siκB-Ras2 were irradiated, and then co-cultured with CFSE-labelled allogeneic T cells for 24 h. The proliferation of T cells was determined by FACS (n=4). E. The efficiency of knockdown IRF4 expression in BMDM via siRNA was determined by qRT-PCR (n=3). Data are shown as mean ± S.E.M. *, $P < 0.05$; **, $P < 0.01$; ***, $P < 0.001$ by unpaired student's t-test (B-D) or one-way ANOVA with Tukey's multiple comparison tests (E).

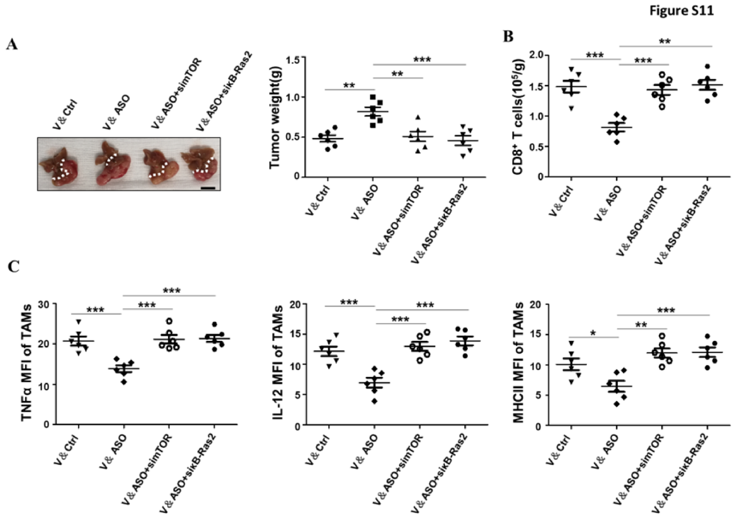

Figure S11. TAM-targeted delivery of simTOR or siκB-Ras2 inhibited miR-99b antagomir-triggered tumour growth. A. Tumours were isolated from HCC-bearing mice at day 29 after different drug treatments, and then were photographed and weighed (n=6). B. The $CD8^+$ T cell number in tumours was analysed and compared by FACS (n=6). C. The MFI of TNFα, IL-12 and MHCII expression in TAMs of HCC-bearing mice was analysed by FACS and compared (n=6). Data are shown as mean ± S.E.M. *, P < 0.05; **, P < 0.01; ***, P < 0.001 by one-way ANOVA with Tukey's multiple comparison tests.

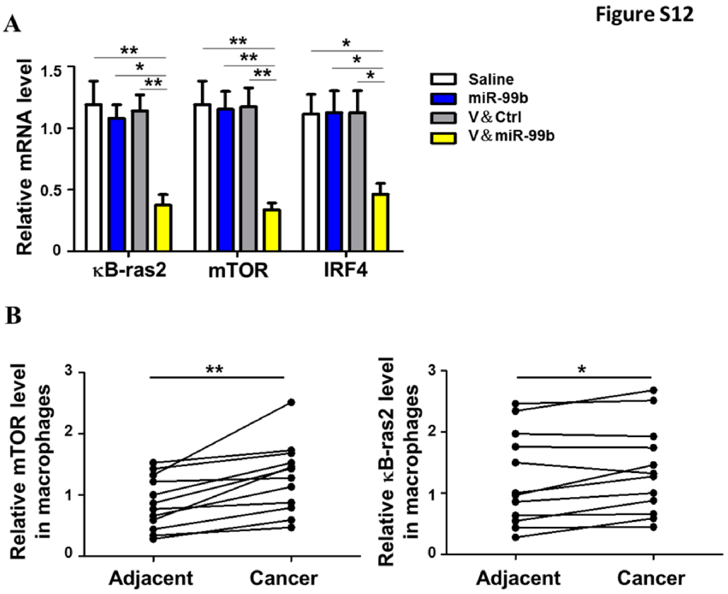

Figure S12. miR-99b/mTOR and/or κB-ras2 axis regulated TAM phenotype both in murine HCC model and liver cancer patients. A. The expression of mTOR, κB-ras2, and IRF4 in TAMs sorted from HCC-bearing mice (related with Figure 1) was measured by qRT-PCR (n=4). B. The expression of mTOR and κB-ras2 in TAMs sorted from cancer and adjacent tissue of liver cancer patients (Figure S3) was detected and compared by qRT-PCR (n=12). Data are shown as mean ± S.E.M. *, P < 0.05; **, P < 0.01 by one-way ANOVA with Tukey's multiple comparison tests (A) or paired student's t-test (B).
