# Supplementary material for: Targeted delivery of miR-99b reprograms tumor-associated macrophage phenotype leading to tumor regression
Source: J Immunother Cancer. 2020 Sep 18;8(2):e000517. doi: 10.1136/jitc-2019-000517 (PMC7511616; doi:10.1136/jitc-2019-000517)
Supplement: Supplementary data [file jitc-2019-000517supp002.pdf]

Additional file 2

Table S1. Clinical data of 12 HCC patients involved in this study

No.	AJCCstage (T stage)	Age	Sex
1	T2	62	M
2	T2	72	M
3	T3	69	M
4	T1	62	M
5	T2	60	M
6	T3	39	M
7	T2	53	M
8	T1	52	F
9	T1	54	M
10	T1	58	M
11	T2	68	F
12	T1	56	M

Table S2. Antibodies and related reagents used in this study

Name	Supplier	Catalog #	Titration
APC CD11b	Biolegend	101212	1/400
Alexa 488 F4/80	Biolegend	123120	1/100
APC F4/80	eBioscience	174801	1/100
Biotin MHC II	BD Pharmingen	553607	1/1000
APC Ly6C	Biolegend	128015	1/200
Biotin Ly6G	Biolegend	127603	1/1000
APC CD3	Biolegend	100236	1/200
Biotin CD3	Biolegend	100303	1/400
Biotin B220	Biolegend	103204	1/400
Biotin Gr1	Biolegend	108403	1/1000
PE CD4	BD Pharmingen	553048	1/200
FITC CD8	BD Pharmingen	553030	1/50
PE CD11c	BD Pharmingen	117307	1/100
V450 CD45	BD Pharmingen	560501	1/800
PE IL-12	eBioscience	127123	1/50
PE NOS2	eBioscience	125920	1/100
PE TNF-a	ebioscience	177321	1/50
APC IL-10	eBioscience	177101	1/50
Rab anti-F4/80	ebioscience	144801	1/400
Biotin CD163	ebioscience	13163982	1/400
PE CD14	ebioscience	12014941	1/200
PE VCAM-1	ebioscience	121061	1/100
Rab anti-MR	Abcam	AB64693	1/1000
Rab anti-ARG1	Abcam	AB91279	1/400
Rab anti-iNOS	Abcam	AB178945	1/400
Rab anti-Ki67	Abcam	AB16667	1/200
Rab anti-STAT3	CST	9139	1/1000
Rab anti-Phospho-STAT3	CST	9145	1/1000
Rab anti-β-actin	CST	4970	1/2000
Rab anti-mTOR	CST	2983	1/1000
Rab anti-IkBα	CST	9242	1/1000
Rab anti-Phospho-IκBα	CST	2859	1/1000
Rab anti- p65	CST	8242	1/1000
Rab anti-Lamin A/C	CST	2032	1/1000
Rab anti-κB-Ras2	Signalway Antibody	24152	1/1000
Mouse anti-α-Tubulin	Santa Cruz Biotechnology	sc-5286	1/1000
Rab anti-IRF4	Santa Cruz Biotechnology	sc-48338	1/1000
PE streptavidin	eBioscience	124317	1/800
Cy3 goat anti-rabbit IgG	Boster Bio Tec	BA1032	1/400

Wang L, *et al. J Immunother Cancer* 2020; 8:e000517. doi: 10.1136/jitc-2019-000517

Table S3. Primers and oligonucleotides used for PCR in this study

Name	Purpose	Sequence
Pri-mir-125a/99b-F	RT-PCR	5'-CTGACGCTTGGCAACCTATTCC
Pri-mir-125a/99b-R	RT-PCR	5'-GCTGTTGAGGTGGTGACAAAGG
miR-99b-5p	RT-PCR	5'-CACCCGTAGAACCGACCTTGCG
miR-125a-5p	RT-PCR	5'-TCCCTGAGACCCTTTAACCTGTGA
IRF4-F	RT-PCR	5'-GGAATTGGTCGAGAGGAG
IRF4-R	RT-PCR	5'-GAGAGCCATAAGGTGCTG
TNF-α-F	RT-PCR	5'-CAGGAGGGAGAACAGAAACTCCA
TNF-α-R	RT-PCR	5'-CCTGGTTGGCTGCTTGCTT
IL-12-F	RT-PCR	5'-GGAAGCACGGCAGCAGAATA
IL-12-R	RT-PCR	5'-AACTTGAGGGAGAAGTAGGAATGG
IL-6-F	RT-PCR	5'-CCACTTCACAAGTCGGAGGCTTA
IL-6-R	RT-PCR	5'-GCAAGTGCATCATCGTTGTTCATAC
IL-10-F	RT-PCR	5'-CCCTTTGCTATGGTGTCCTT
IL-10-R	RT-PCR	5'-TGGTTTCTCTTCCCAAGACC
Arg1-F	RT-PCR	5'-AGACAGCAGAGGAGGTGAAGAG
Arg1-R	RT-PCR	5'-CGAAGCAAGCCAAGGTTAAAGC
MR-F	RT-PCR	5'-AAACACAGACTGACCCTTCCC
MR-R	RT-PCR	5'-GTTAGTGTACCGCACCCTCC
Actin-F	RT-PCR	5'-CATCCGTAAAGACCTCTATGCCAAC
Actin-R	RT-PCR	5'-ATGGAGCCACCGATCCACA
mTOR F	RT-PCR	5'-ACCGGCACACATTTGAAGAAG
mTOR R	RT-PCR	5'-CTCGTTGAGGATCAGCAA GG
κB-Ras2 F	RT-PCR	5'-ACCTCAGCGGGAGTGCT
κB-Ras2 R	RT-PCR	5'-GTC CTC CTG GGTCTCGATCA
human-κB-Ras2 F	RT-PCR	5'-TGTAGTGGGTTCGGAGATGAT
human-κB-Ras2 R	RT-PCR	5'-CGGTCTGTCTCAATGGAGCC
human-mTOR F	RT-PCR	5'-TCCGAGAGATGAGTCAAGAGG
human-mTOR R	RT-PCR	5'-CACCTTCCACTCCTATGAGGC
mTOR F	PCR	5'-GCGAATTCCTGAGGCCTGGAAAACCACGTCGT
mTOR R	PCR	5'-GCCTGCAGCGTGGTGTCTAGATGTGGCTTTAC
κB-Ras2 F	PCR	5'-GCCATATGGACTAGGTACTGTAAGCATAC
κB-Ras2 R	PCR	5'-TGCTGCAGGATAAGCTCACTTGATAGCAC
mTOR mut F	PCR	5'-CTG AGA AAA CGC ACA TTT GAC TTA
mTOR mut R	PCR	5'-TAA GTC AAA TGT GCG TTT TCT CAG
Nkiras2 mut F	PCR	5'-CTA GAG CCT TATCAAGCGCTGT
Nkiras2 mut R	PCR	5'-ACA GCG CTT GATAAGGCTCTAG
IRF4 siRNA1	siRNA	5'-CCTCTCATTCTTTAGAATTGTT
IRF4 siRNA2	siRNA	5'-GCTTCACAAGGAAAGCTGTGTG
IRF4 siRNA3	siRNA	5'-GGACACACCUAUGAUGUUATT
mTOR siRNA1	siRNA	5'-CCACCAGAATTGGCAGATT
mTOR siRNA2	siRNA	5'-GCAAAGACCTCATGGGCTT
mTOR siRNA3	siRNA	5'-GCCAATCATTCGCATTCAA

Wang L, *et al. J Immunother Cancer* 2020; 8:e000517. doi: 10.1136/jitc-2019-000517

κB-Ras2 siRNA1	siRNA	5'-CCCTCCTAGAGCCTTTCAT
κB-Ras2 siRNA2	siRNA	5'-GGAGCAGGTGCGTTTCTAT
κB-Ras2 siRNA3	siRNA	5'- CCGGGAGGTTGAAAGCTAA

*F, forward; R, reverse.
